# Identification of Small Open Reading Frame-Encoded Peptides in Glioma by an Optimized Proteomics Strategy

**DOI:** 10.1016/j.mcpro.2025.101016

**Published:** 2025-06-11

**Authors:** Tingting Zhang, Jian Cheng, Jiao Li, Zixia Ye, Na Li, Jifeng Wang, Xiaojuan Yang, Yong Peng

**Affiliations:** 1 Center for Molecular Oncology, Frontiers Science Center for Disease-related Molecular Network, State Key Laboratory of Biotherapy and Cancer Center, West China Hospital, Sichuan University, Chengdu, China; 2 Department of Neurosurgery West China Hospital, Sichuan University, Chengdu, China; 3 Laboratory of Protein and Peptide Pharmaceuticals & Laboratory of Proteomics, institute of Biophysics, Chinese Academy of Sciences, Beijing, China

**Keywords:** small open reading frame-encoded peptides (SEPs), SEP enrichment, glioma, mass spectrometry

## Abstract

Small open reading frame–encoded peptides (SEPs), translated from previously unannotated genomic regions, have emerged as important regulators in diverse physiological and pathological processes. While ribosome profiling and bioinformatics analysis can predict putative SEPs, mass spectrometry (MS) is the only method for their definitive identification. However, MS-based SEP detection faces significant challenges due to SEP’s short length and low abundance. To address these limitations, we developed an ammonium formate-mediated C8 solid-phase enrichment (AmF-C8-SPE) strategy that significantly outperforms classic C8-SPE, yielding superior SEP identification with enhanced unique peptide ratios and sequence coverage. By coupling AmF-C8-SPE with fractionation and LC-MS/MS analysis of glioma samples from 18 patients, we identified 549 novel SEPs, 113 of which exhibited differential expression between tumors and adjacent normal tissues. Importantly, randomly selected SEPs were validated by MS spectral matching with synthetic peptides and by confirming recombinant fusion protein expression in cells. Furthermore, Mfuzz clustering and ROC curve analyses revealed SEPs associated with glioma progression. DeepLoc-based prediction followed by confocal microscopy imaging confirmed nuclear localization of two candidate SEPs (IP_613981 and SPROHSA206836). Therefore, this study establishes an optimized SEP identification approach and the first comprehensive SEP profiling in glioma, providing a valuable resource to discover novel glioma biomarker and therapeutic target.

## Highlights

• An optimized method termed AmF-C8-SPE was developed for SEP enrichment.
• Coupling AmF-C8-SPE with fractionation and LC-MS/MS identified 549 SEPs in glioma.
• Mfuzz clustering and ROC analysis revealed SEPs associated with glioma progression.
• Two SEPs (IP_613981 and SPROHSA206836) were confirmed to localize in the nucleus.

## Graphical Abstract

**Figure:**
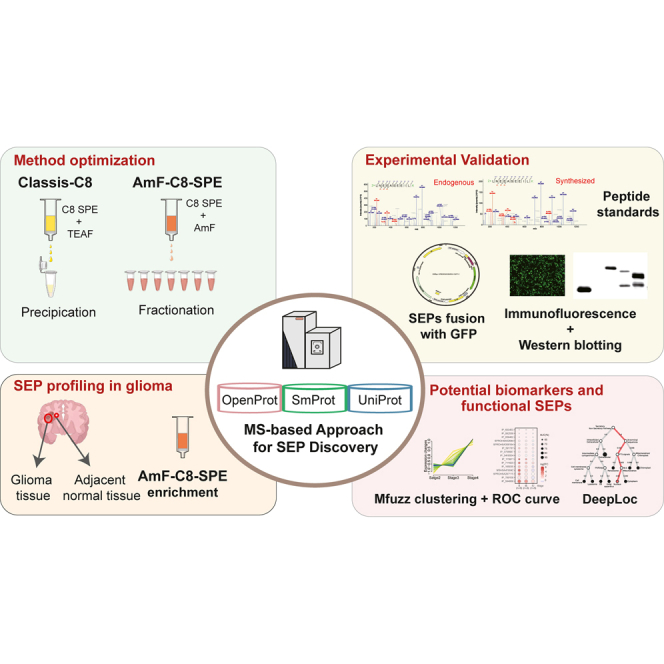

## In Brief

This study developed an optimized proteomics workflow integrating ammonium formate -mediated C8 solid - phase enrichment (AmF-C8-SPE) with fractionation and LC-MS/MS analysis to significantly improve small open reading frame–encoded peptide (SEP) identification. Compared to classic C8 solid-phase enrichment (Classic-C8), AmF-C8-SPE demonstrated superior SEP identification efficiency and data quality. Applying this approach to clinical glioma samples, we identified 549 novel SEPs, including 113 with differential expression between tumors and adjacent normal tissues, establishing the first comprehensive qualitative and quantitative SEP profiling in glioma.

Short open reading frame-encoded peptides (SEPs), also known as microproteins, are typically translated from small open reading frames (sORFs) less than 300 nucleotides in length. Historically overlooked in genome annotations due to their short sequences and the stringent criteria of conventional gene prediction algorithms (1, 2), SEPs have gained increasing recognition with advances in sequencing and mass spectrometry (MS) technologies. These SEPs originate from diverse genomic regions, including the untranslated region (UTR) of mRNA (3), ribosomal RNA (4), pri-miRNA (5), long non-coding RNA (lncRNA) (6), and circular RNA (7). Increasing studies demonstrate their functional significance across multiple biological processes, such as modulating autoimmune responses through protein–protein interactions (8), regulating gene expression post-transcriptionally (9, 10), facilitating embryonic development (11, 12), and influencing tumor progression as oncogenic or tumor suppressive factors (13, 14). Despite the abundance of predicted sORFs in genomes, only a limited number of SEPs have been characterized, highlighting the urgent need for more efficient SEP identification methods.

Current approaches for SEP identification include high-throughput sequencing with bioinformatics analysis, ribosome profiling, and mass spectrometry. While computational and sequencing methods can predict numerous SEPs, MS is the fold standard for definitive identification, providing direct evidence of SEP presence in biological samples with high specificity (15). However, MS detection efficiency remains limited, with less than 0.3% of predicted SEPs being detected (16). This technical challenge is largely due to SEP’s small size and low abundance, necessitating effective enrichment strategies prior to MS analysis. Several SEP enrichment methods have been reported, including organic solvent/acid precipitation, C8/C18 solid-phase enrichment (SPE), 30-kDa molecular weight cut-off (MWCO) filtration, size exclusion chromatography (SEC), and Fe_3_O_4_@SiO_2_/TiO_2_ - based approaches (17, 18, 19, 20, 21, 22, 23). Among these, C8 SPE has proven particularly effective for SEP enrichment (19). Here, we developed an optimized ammonium formate-mediated C8 solid-phase enrichment (AmF-C8-SPE) method that significantly improves SEP enrichment efficiency compared to conventional C8-SPE.

Gliomas represent the most prevalent and aggressive brain tumors (24). Current standard treatments for glioma involve surgical resection followed by adjuvant radiotherapy and chemotherapy (25). However, malignant gliomas, particularly glioblastomas, exhibit poor prognosis due to their rapid proliferation, diffuse invasion, and significant genetic heterogeneity (26). These clinical challenges underscore the critical need to identify novel diagnostic/prognostic biomarkers and therapeutic targets. Emerging evidence implicates SEPs as key regulators in glioma pathogenesis. For example, the circ-E-Cad-encoded SEP promotes glioma progression via EGFR-STAT3 pathway activation (27), while the SEP encoded by the lncRNA FOXD1-AS1 serves as a glioma-specific biomarker and holds potential as a therapeutic target (28). These discoveries highlight the functional significance of SEPs in glioma progression, motivating further exploration of glioma-associated SEPs.

In this study, we optimized the C8 SPE method for SEP enrichment and developed a new approach that significantly improved MS-based SEP discovery, achieving enhanced SEP identification yield, increased proportion of unique peptides, and superior sequence coverage compared to conventional approaches. Applying this improved method, we identified and quantified 549 novel SEPs in 18 pairs of glioma tumors and adjacent normal tissues. Moreover, randomly selected SEPs were validated by MS-based verification (synthetic peptide matching) and cellular biology experiments (fusion protein expression). Subsequent bioinformatics analysis revealed SEPs associated with glioma progression, and nuclear localization of selected SEPs were experimentally confirmed. Therefore, this study establishes a powerful proteomic approach to identify SEPs and provides a valuable resource to discover novel glioma biomarkers and therapeutic targets.

## Experimental Procedures

### Experimental Design and Statistical Rationale

In this study, we systematically evaluated different SEP enrichment methods: Classic-C8, AmF-C8-SPE, AmF-C8-SPE (fractions = 4), and AmF-C8-SPE (fractions = 7). The experimental workflow includes five critical steps: (1) protein extraction from biological samples, (2) SEP enrichment, (3) LC-MS/MS sample preparation, (4) LC-MS/MS analysis, and (5) bioinformatics data analysis. To evaluate the reproducibility of these different methods, we performed three technical replicates using HEK293 T samples. For experiments of clinical samples, we initially collected 52 samples (26 glioma tumors and their adjacent normal tissues). Due to incomplete clinical information of some samples and considering balanced representation across sample stages, 40 samples (20 pairs) were enrolled for SEP enrichment and subsequent label-free quantitative proteomic analysis. The cohort consists of nine pairs of Stage II, five pairs of Stage III, and six pairs of Stage IV samples. During the initial raw data analysis, two pairs of samples were excluded due to failure of quality control using the "Minora Feature Detector" module of Thermo Scientific Proteome Discoverer 2.4, yielding 18 qualified pairs (Stage II, n = 9; Stage III, n = 3; Stage IV, n = 6) for subsequent discovery of SEPs by proteomic profiling. Pearson or Spearman correlation analysis was performed using the Performance Analytics package in R. The correlation coefficient (r) reflects the strength of the relationship, with values greater than 0.8 indicating an extremely strong correlation. Two-group comparisons were analyzed using two-tailed Student’s *t*-test in GraphPad Prism v.9.0. with statistical significance thresholds set at ∗*p* < 0.05, ∗∗*p* < 0.01 and ∗∗∗*p* < 0.001.

### Declaration of Helsinki Principles

Glioma samples were collected at West China Hospital of Sichuan University with the approval of the institutional research ethics committee (Approval No. 2019(541)). Informed consent was obtained from participants or their families. The study was conducted in accordance with the ethical principles outlined in the Declaration of Helsinki.

### Glioma Sample Collection

To minimize blood contamination and prevent protein degradation, tissue samples were promptly rinsed with cold saline solution, flash-frozen in liquid nitrogen, and stored at −80 °C for future analysis. Adjacent noncancerous tissues were typically located 1 to 2 cm from tumor margins. Clinical information for all patients is provided in Supplemental Table S1.

### Cell Lines and Cell Cultures

Human embryonic kidney 293T (HEK293T) cell, human lung cancer cell H1299, and human glioma cell U251 were cultured in DMEM medium (Gibco #C11995500BT) supplemented with 10% fetal bovine serum and 1% penicillin/streptomycin (Biosharp #P1400–100). Cells were maintained at 37 °C in a 5% CO_2_ incubator.

### Protein Extraction and SEP Enrichment

For the Classic-C8 method, SEPs were enriched following procedures described in previous studies (19, 20). ∼ 1 × 10ˆ7 cells were lysed in 500 μl of lysis buffer (50 mM HCl, 0.1% β-mercaptoethanol, 0.05% Triton X-100) containing protease inhibitor cocktail tablets for 30 min at room temperature. Following centrifugation at 20,000×*g* for 20 min at 4 °C, the supernatant from 3 mg of cell lysates was filtered through a 5-μm syringe filter (Millipore # SMWP01300) and collected. Subsequently, Bond Elute C8 silica cartridges (Agilent #12102002) were conditioned with one column volume of methanol and two column volumes of triethylammonium formate (TEAF) buffer (pH 3.0) before applying the lysate. Enriched SEPs were eluted sequentially with two column volumes of 75% acetonitrile (ACN) in TEAF buffer. The eluted fractions were concentrated to less than 100 μl by vacuum freeze drying. Finally, enriched SEPs were precipitated with chloroform/methanol to remove residual detergent, and the precipitate was frozen to dry and dissolved in 8 M urea/100 mM Tris-HCl, pH 8.5.

For our optimized method, AmF-C8-SPE, ∼1 × 10ˆ7 cells were suspended in 500 μl of lysis buffer (8 M urea/100 mM Tris-HCl, pH 8.5) containing Protease Inhibitor Cocktail Tablets (Roche) and subjected to sonication on ice for 2 min. In whole tissue protein extraction, ∼20 mg of tissue was cut into small pieces, homogenized in 500 μl of the same lysis buffer, and then homogenized for about 1 min followed by sonication on ice for 2 min. The supernatant was carefully collected after centrifugation at 20,000×*g* at 4 °C for 20 min, and 3 mg cell lysate and 1 mg tissue lysate were employed for the enrichment of SEP. After the supernatant was filtered through a 5-μm syringe filter (Millipore # SMWP01300) and collected. Before inputting the total lysate, the C8 cartridge (Agilent #12102002) was activated with one column volume of methanol and equilibrated with two column volumes of ammonium formate (AmF) buffer (pH 3.0). SEPs were then enriched using one column volume of 75% ACN in AmF buffer. Our optimized method employs AmF buffer with C8 cartridge column to enrich SEPs and is designated as AmF-C8-SPE. For the gradient elution, SEPs were sequentially eluted using 1 ml 15%, 20%, 25%, 30%, 35%, 40% and 75% ACN or 20%, 40%, 60% and 75% ACN in AmF buffer. The eluted fractions were frozen to dry and dissolved in 20 μl of 8 M urea/100 mM Tris-HCl, pH 8.5.

### Tricine Gel Analysis of Enriched SEP Samples

The enriched SEPs were dissolved in 5 × loading buffers containing 10% SDS, 500 mM DTT, 50% glycerol, 250 mM Tris-HCl (pH 6.8), and 0.1% bromophenol blue. After denaturation at 95 °C for 10 min, the samples were loaded onto a 10% Tricine-glycine SDS-PAGE gel (29) and electrophoresed at 60 V for 30 min, followed by 100 V for 90 min. The gel was stained using Coomassie Brilliant Blue and subsequently destained with a solution containing 25% ethanol, 8% acetic acid, and 67% water.

### Sample Digestion for MS

Proteins/SEPs were reduced with 10 mM DTT for 1 h at 37 °C, alkylated with 20 mM IAM for 45 min in the dark at room temperature, and then digested overnight at 37 °C with trypsin at a final concentration of 0.01 μg/μl (Promega) in less than 1 M urea/50 mM Tris-HCl (pH 8.0). Formic acid (FA) was added to the digested samples at a final concentration of 0.1% to terminate the reaction. Subsequently, after desalting by C18 ZipTips (Millipore #ZTC18S096), the peptides were dried by a vacuum freeze dryer and subjected to the following LC-MS/MS analysis.

### LC–MS/MS Analysis

Peptide samples (500 ng each) were reconstituted in 0.1% FA/H_2_O and subjected to LC-MS/MS analysis using an Easy-nLC 2000 HPLC coupled with an Orbitrap Exploris 480 mass spectrometer (Thermo Fisher Scientific). Peptides were chromatographically separated using a C18 column (75 μm × 25 cm) packed with Reprosil-Pur C18 AQ particles (1.9 μm, Dr Maisch HPLC GmbH) and eluted with a gradient of solvent A (0.1% formic acid) and solvent B (acetonitrile/0.1% formic acid). The flow rate was set at 300 nl/min with the following gradient: 3% B (0 min), 8% B (5 min), 25% B (55 min), 38% B (69.5 min), 95% B (71 min), and 95% B (78 min).

For MS analysis, the Orbitrap Exploris 480 mass spectrometer was operated in data-dependent acquisition mode (DDA). Each MS1 spectrum was acquired at a resolution of 60,000 (at m/z 200) within a 350 to 1500 m/z scan range. The normalized automatic gain control (AGC) target was 300% with a custom maximum injection time. MS/MS spectra were acquired at a resolution of 15,000 (at m/z 120) with an isolation window of 1.6 m/z and a 75% normalized AGC target with a custom maximum injection time.

### Identification and Quantification of Annotated Proteins and SEPs

The LC–MS/MS raw data were analyzed using Thermo Scientific Proteome Discoverer 2.4 with the SEQUEST HT engine. The analysis utilized the following databases: (1) Human UniProt database, which includes the Human_SwissProt (20,440 entries, downloaded from UniProt on January 3, 2024) and the Human_TrEMBL (187,523 entries, downloaded from UniProt on September 20, 2023); (2) Human SEP database from SmProt V2.0, containing 433,032 entries; and (3) Human SEP database from OpenProt, which comprises 593,496 entries.

The raw data were searched against the UniProt and SmProt databases when comparing different methods, while glioma raw data were searched against a merged database comprising UniProt, SmProt, and OpenProt. Trypsin was specified as the enzyme, allowing for a maximum of two missed cleavages. The precursor mass tolerance was set to 10 ppm, and the product ion tolerance to 0.02 Da. Carbamidomethylation of cysteine was designated as a fixed modification, while methionine oxidation and N-terminal acetylation were considered variable modifications. False discovery rate (FDR) analysis was conducted using the Percolator algorithm, and peptides with an FDR <1% were classified as high-confidence peptides.

For label-free quantification (LFQ) analysis, we employed Thermo Scientific Proteome Discoverer 2.4 to perform quantification, normalization, and missing value imputation. First, all MS raw data were analyzed using the Processing Workflows and the resultant.msf files were subjected to Consensus Workflows analysis. During the Processing Workflows, chromatographic peaks required ≥5 data points across the elution profile, and LC-MS peak co-elution was constrained to a 0.2-min retention time window using the Minora feature detector node to ensure spectral fidelity. The Consensus Workflows comprised the following steps (1): retention time alignment utilizing high-confidence identified peptides (2); noise reduction by application of signal-to-noise ratio (S/N) threshold filter (3); precursor ion intensity-based quantification (4); normalization on total peptide abundance (5); missing value imputation through replicate-based low-abundance resampling to address stochastic ion suppression effects.

## Bioinformatics Analysis of SEPs

The data were processed using R (version 4.3.0). Differential protein/SEP analysis was conducted using the DESeq2 package. Proteins/SEPs with *p*-value ≤0.05 and log2 |fold change (FC)| ≥ 1 were classified as up- or downregulated, respectively. Pathway analysis of annotated proteins was performed using the aPEAR package.

Considering the individual variability among patients with tumors, we first calculated the fold change for each SEP by dividing its abundance in tumor tissue by that in adjacent normal tissue. Subsequently, we conducted Mfuzz clustering analysis and ROC curve analysis to calculate AUC values across the 18 paired samples. Mfuzz clustering analysis of SEPs was carried out with the Mfuzz package. The AUC value of SEPs was analyzed by Wei Sheng Xin (https://www.bioinformatics.com.cn/plot_basic_one_or_multi_ROC_curve_plot_106) based on the pROC package (30).

### Subcellular Location Analysis of SEPs

The FASTA file containing all SEPs with corresponding amino acid sequences was compiled for subcellular localization prediction. This dataset was subsequently uploaded to the DeepLoc-1.0 platform (https://services.healthtech.dtu.dk/services/DeepLoc-1.0/) and analyzed by initiating the "Submit" function. Upon completion of the computational analysis, the results were exported as a CSV file for downstream processing. Subcellular localization percentages across all SEPs were calculated using Microsoft Excel. For hierarchical tree visualization, the DeepLoc-1.0 analysis module was employed, while predicted signal visualization was conducted using the enhanced DeepLoc-2.0 platform (https://services.healthtech.dtu.dk/services/DeepLoc-2.0/). These methodological details were formally incorporated into the Experimental Procedures section of the study.

### MS-Based SEP Validation

To validate the identification of SEPs by MS, 10 standard peptides corresponding to 10 SEPs were synthesized by Syn High Quality Peptide. These standard peptides were dissolved in ddH_2_O to prepare a peptide mixture with a final concentration of ∼500 pmol/μl for each peptide. The peptide mixture was then analyzed using the same LC–MS/MS methods as previously mentioned.

### Plasmid Constructs and Cell Transfection

The SEP and GFP fusion protein vector (SEP-EGFPmut) was constructed by cloning the 5′ UTR and ORF of the SEP into the pEGFPmut-N1 vector. For transcripts lacking the 5′ UTR, only the full-length ORF was cloned into the pEGFPmut-N1 vector. The fusion construct incorporates a 13-amino-acid linker (∼1.4 kDa) between the GFP and SEP sequences. The start codon of GFP (ATGGTG) was mutated to ATTGTT (pEGFPmut). The empty pEGFPwt-N1 (EGFPwt) and pEGFPmut-N1 (EGFPmut) vectors were used as controls. Primer sequences for vector construction were listed in Supplemental Table S2. HEK293T cells were transfected with the EGFPwt, EGFPmut, and SEP-EGFPmut plasmids using Lipofectamine 2000 (Invitrogen #11668–019) according to the manufacturer's instructions.

### Western Blotting

Cells were lysed in RIPA buffer (Beyotime #P0013B) supplemented with protease and phosphatase inhibitors (Roche #PPC1010), and protein concentrations were determined using the BCA Protein Assay Kit (Beyotime #P0009). Equal amounts of protein were separated on 10% SDS-PAGE gels and transferred onto 0.45 μm PVDF membranes (GE #10600021). The membrane was then incubated overnight at 4 °C with GFP or vinculin (Proteintech #26520-1-AP) primary antibodies, followed by incubation with secondary antibodies for 1.5 h at room temperature. After three washes with 0.1% TBS-T buffer, the membrane was incubated with an enhanced chemiluminescent horseradish peroxidase (HRP) substrate (Thermo Fisher Scientific #34076) and visualized using the ChemiDoc XRS + System (Bio-Rad).

### Confocal Microscopy Assays

HEK293T cells were transfected with different plasmids and cultured for 24 h. The confocal dishes were pre-coated with poly-D-lysine (PDL) coating (0.1 mg/ml in sterile PBS) by incubation at 37 °C for 6 h. Transfected cells were subsequently plated on these confocal dishes and cultured for another 24 h s After the nuclei were stained with Hoechst solution, cells were fixed with 4% paraformaldehyde and subjected to visualized GFP and Hoechst fluorescence under laser confocal microscopy (Leica Stellaris, Germany).

## Results

### Optimized Enrichment and MS-Based Workflow for Improved SEP Discovery

**Fig. 1:**
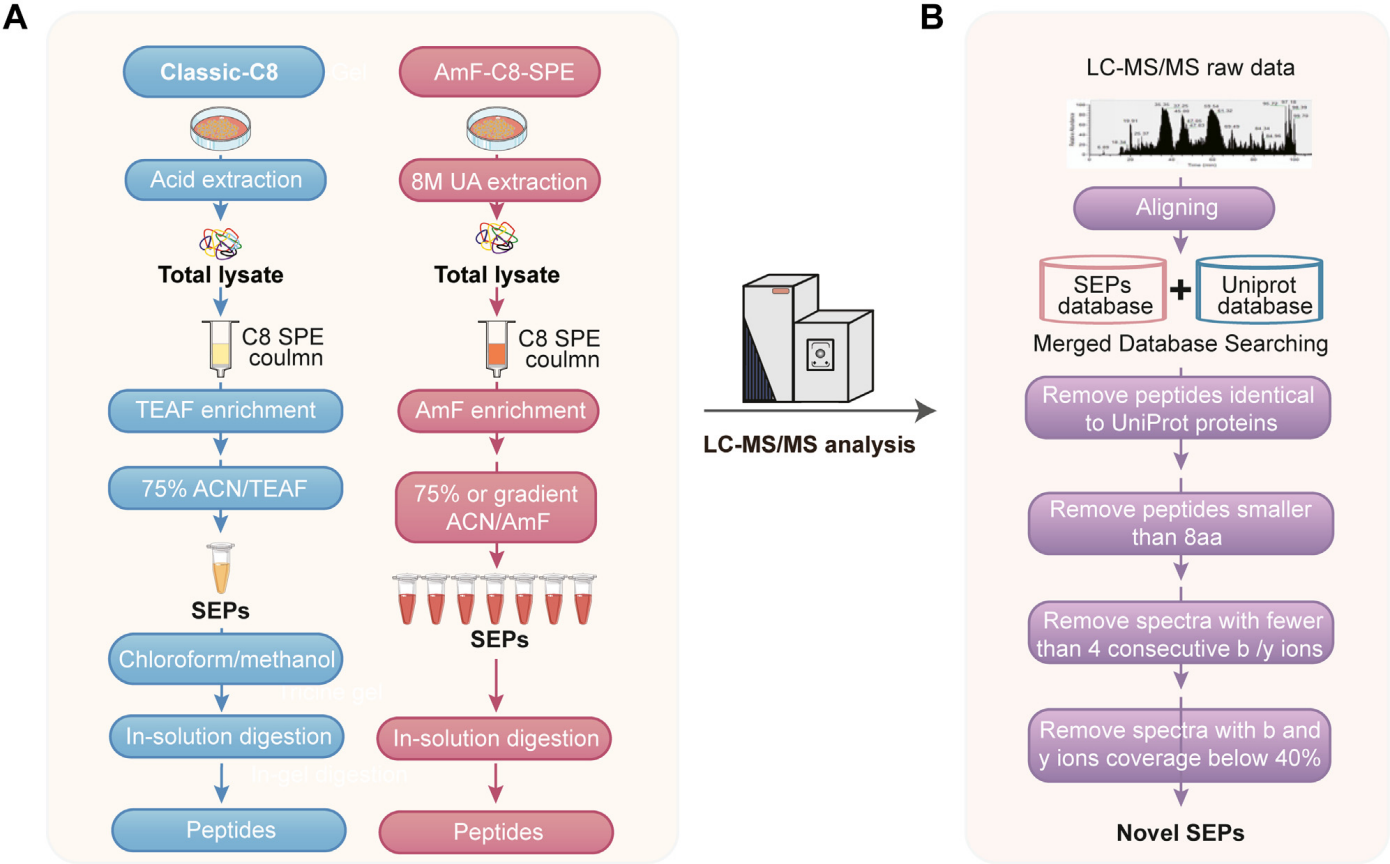
**Schematic illustration of the workflow for MS-based SEP discovery**. *A*, the workflow including Classic-C8 and AmF-C8-SPE for SEP enrichment from cells. *B*, MS-based identification and quantification of SEPs. ACN, acetonitrile; AmF, ammonium formate; AmF-C8-SPE, ammonium formate - mediated C8 solid - phase enrichment; Classic-C8, classic C8 solid - phase enrichment (C8 SPE); LC-MS/MS, liquid chromatography - tandem mass spectrometry; SEPs: small open reading frame - encoded peptides; TEAF, triethylammonium formate.

Based on established C8 SPE and fractionation approach for SEP enrichment (17, 19, 31), we developed an optimized ammonium formate-mediated C8 solid-phase enrichment (AmF-C8-SPE) method to significantly improve SEP enrichment and identification. Figure 1*A* depicts the workflows of our novel AmF-C8-SPE method as well as the classic C8 SPE (Classic-C8) approach. In the Classic-C8 method, triethylammonium formate (TEAF) buffer was used to enrich SEPs after the C8 column adsorption and 75% ACN/TEAF buffer elution. Since TEAF is incompatible with MS, it is removed by methanol/chloroform precipitation (19). The AmF-C8-SPE method uses MS-compatible ammonium formate (AmF) buffer instead of TEAF buffer to eliminate the need for precipitation. To reduce sample complexity and enhance SEP enrichment, the fixed concentration of ACN/TEAF used in traditional methods is replaced with a gradient ACN/AmF buffer to achieve fractional separation in our AmF-C8-SPE method. Subsequently, the enriched samples undergo trypsin digestion, LC-MS/MS analysis, and comprehensive bioinformatic processing to identify both annotated and novel SEPs.

Database selection and stringent filtering criteria are implemented to ensure robust SEP identification. Given that SmProt and OpenProt are the most comprehensive and publicly available SEP databases (32, 33), our MS data were searched against both databases. To maintain high confidence in our results, MS-detected SEPs were subjected to rigorous manual spectral inspection (20, 34). Peptides meeting any of the following criteria were excluded from the list of detected novel SEPs: (1) identical peptide sequence to any annotated UniProt protein/peptide; (2) length less than eight amino acids; (3) spectra containing fewer than four consecutive b or y ions; and (4) spectra with less than 40% coverage of b and y ions (Fig. 1*B*).

### AmF-C8-SPE With Fractionation Represents a More Efficient Method to Identify SEPs

**Fig. 2:**
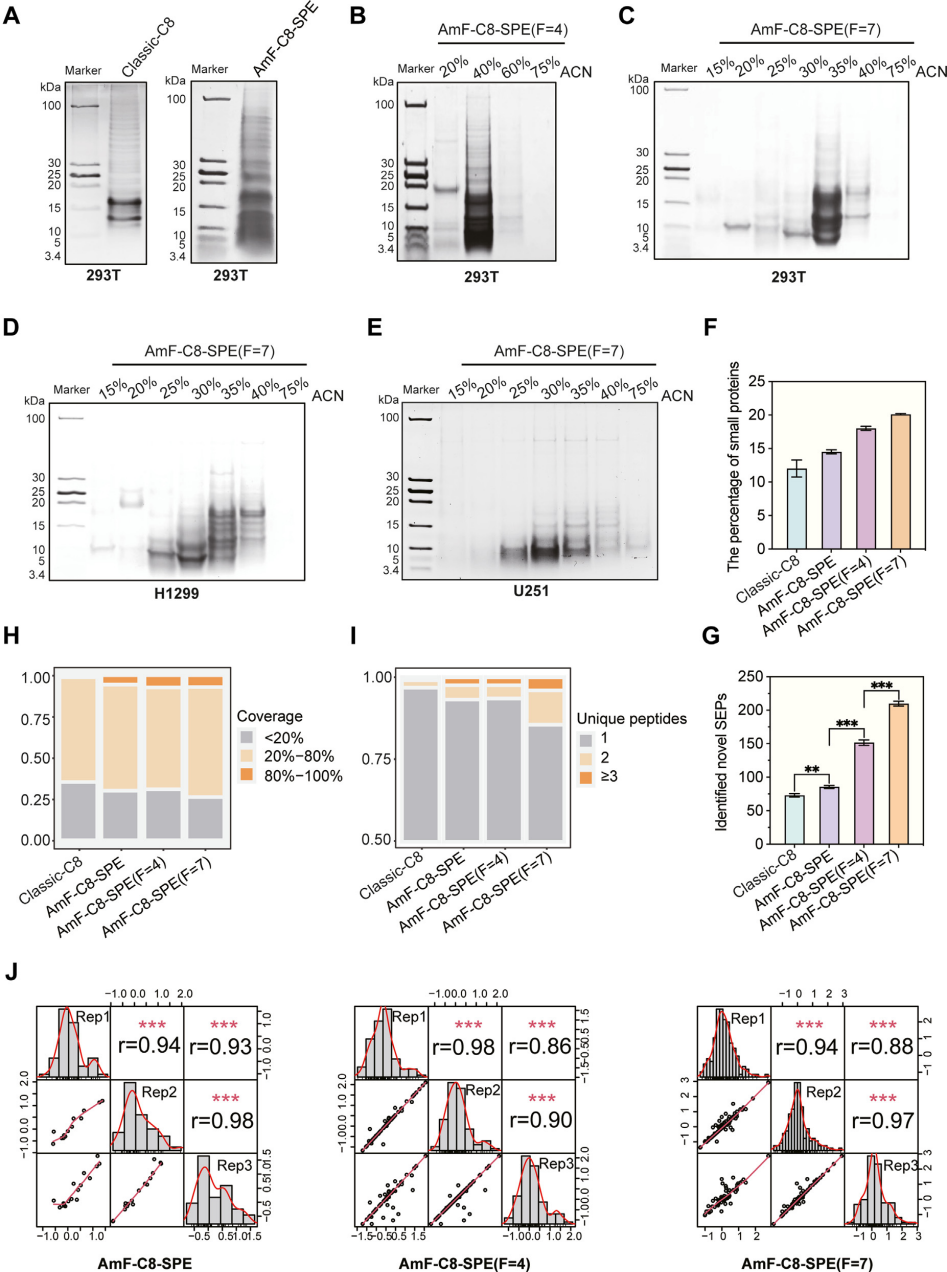
**Comparison of different approaches for SEP discovery.** Tricine SDS-PAGE analysis of enriched SEPs using (*A*) Classic-C8 and AmF-C8-SPE, (*B*) AmF-C8-SPE (fractions = 4), and (*C*) AmF-C8-SPE (fractions = 7) methods. (*D*) and (*E*) Tricine SDS-PAGE analysis of enriched SEPs from H1299 cells and U251 cells using AmF-C8-SPE (fractions = 7) methods. *F*, the percentage of small protein (≤100 aa) and (G) the number of SEPs identified using Classic-C8, AmF-C8-SPE, AmF-C8-SPE (fractions = 4), and AmF-C8-SPE (fractions = 7) methods. *∗∗p* ≤ 0.05, *∗∗∗p* ≤ 0.001. *H*, the percentage of coverage and (*I*) unique peptides in SEPs identified by Classic-C8, AmF-C8-SPE, AmF-C8-SPE (fractions = 4), and AmF-C8-SPE (fractions = 7). *J*, Pearson’s correlation analysis of SEPs quantified in three independent replicates using AmF-C8-SPE, AmF-C8-SPE (fractions = 4), and AmF-C8-SPE (fractions = 7) methods. AmF-C8-SPE, ammonium formate-mediated C8 solid-phase enrichment; Classic-C8, C8 solid-phase enrichment (C8 SPE); F, fractions; SEPs, small open reading frame-encoded peptides.

Tricine SDS-PAGE analysis, a well-established method for assessing the enrichment efficiency of small proteins (29), was utilized to compare SEP enrichment between Classic-C8 and AmF-C8-SPE with or without fractionation. The Classic-C8 method primarily enriched small peptides/proteins within a limited molecular weight (MW) range of 12 to 17 kDa (Fig. 2*A*, right). In contrast, our optimized AmF-C8-SPE method demonstrated superior performance, enriching a broader MW range of 3.4 to 19 kDa (Fig. 2*A*, left and Fig. 2*B*, *C*). These results indicate that AmF-C8-SPE significantly enhances SEP enrichment efficiency relative to the Classic-C8 method.

To optimize the elution conditions for AmF-C8-SPE, we evaluated two gradient schemes: 4-fraction (Fig. 2*B*) and 7-fraction (Fig. 2*C*) elution protocols. SEPs from HEK293T cell lysates were predominantly enriched at 40% ACN/AmF using the 4-fraction scheme and at 35% ACN/AmF with the 7-fraction approach. Notably, application of the 7-fraction method to other two types of cell lines (H1299 and U251) revealed distinct elution profiles, with SEPs distributed across multiple gradient concentrations (25%, 30%, 35%, and 40% ACN/AmF) rather than concentrating in a single fraction (Fig. 2*D*, *E*). These results indicate that the elution gradient scheme can be optimized for different sample types to maximize SEP enrichment.

LC-MS/MS detection and the follow-up database retrieval further confirmed the improvement of AmF-C8-SPE in enriching small proteins/peptides. Quantitative assessment revealed that low-molecular-weight species (≤100 aa) constituted 14.51%, 18.02% and 20.12% of total identified proteins/peptides in single-shoot, 4-fraction, and 7-fraction AmF-C8-SPE approaches, respectively, compared to only 12.03% with the Classic-C8 method (Fig. 2F). Meanwhile, AmF-C8-SPE identified 86 SEPs, surpassing 73 SEPs identified by Classic-C8. Moreover, the 4-fraction and 7-fraction AmF-C8-SPE approaches identified 152 (∼2-fold increase) and 210 (∼3-fold increase) SEPs, respectively (Fig. 2*G*). Notably, AmF-C8-SPE using either elution gradient scheme achieved substantially improved sequence coverage (≥80%) and identification confidence (≥3 unique peptides) compared to Classic-C8 (Fig. 2*H*, *I*). Furthermore, AmF-C8-SPE achieved good reproducibility for identified SEPs, with Pearson correlation coefficients >0.86 for technical replicates (Fig. 2*J*). Together, these results indicate that AmF-C8-SPE with fractionation is a substantially more effective approach for SEP enrichment and characterization.

### SEP Profiling in Glioma

**Fig. 3:**
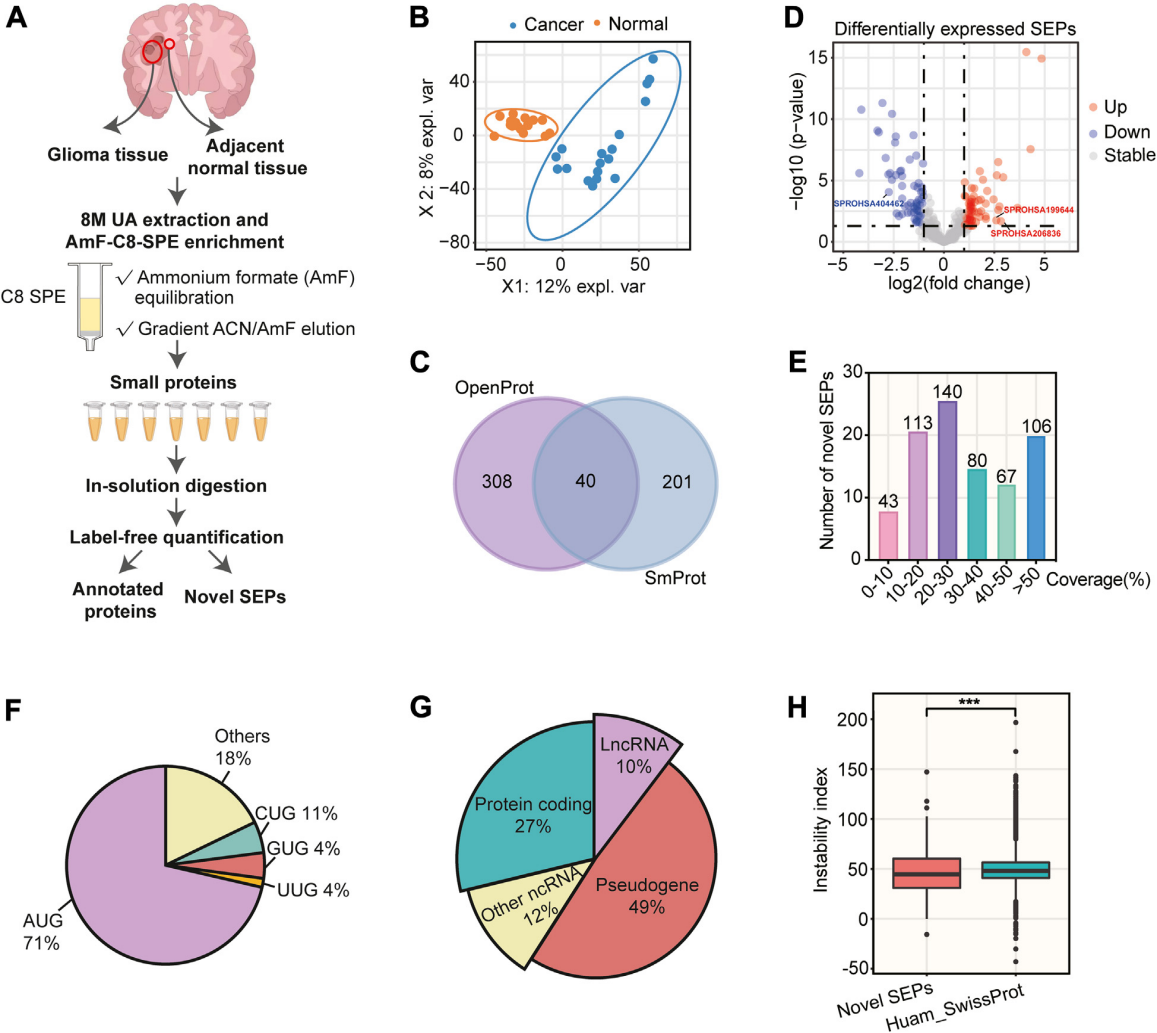
**Quantitative proteomic profiling of SEPs in glioma**. *A*, The workflow of SEP discovery in glioma tumors and adjacent normal tissues. *B*, partial least squares-discriminant analysis of identified proteins from glioma tumors and normal tissues. *C*, Venn diagram of SEPs identified in glioma tissues using OpenProt and SmProt databases. *D*, Volcano plot of differentially expressed SEPs between glioma tumors and normal tissues. *Red*, *blue*, and *gray dots* represent the upregulated, downregulated, and stable SEPs, respectively. (*p*-value ≤0.05; log2|fold change (FC)| ≥ 1). *E*, coverage distribution of identified SEPs. *F*, Start codon analysis of identified SEPs. *G*, the transcript classification of identified SEPs. *H*, the stability analysis of identified SEPs and canonical proteins in the Human_SwissProt database. AmF-C8-SPE, ammonium formate - mediated C8 solid-phase enrichment; C8 SPE, C8 solid-phase enrichment; lncRNA, long noncoding RNA; SEPs, small open reading frame-encoded peptides.

Having validated the enhanced performance of AmF-C8-SPE for SEP enrichment, we employed this optimized method coupled with label-free quantitative MS to profile SEPs in 18 pairs of glioma tumors and adjacent normal tissues (Fig. 3*A*). Tricine SDS-PAGE analysis showed distinct elution profiles between tissue types: SEPs from normal tissues were primarily distributed across the 20%, 25%, 30%, and 35% ACN/AmF gradients, while those from tumor tissues were eluted in the 25%, 30%, 35%, and 40% ACN/AmF gradients (Supplemental Fig. S1*A*). Consequently, we selected AmF-C8-SPE with seven fractions for all glioma samples. In LC-MS/MS analysis, a quality control sample was included for every pair of glioma samples to assess data quality. The result showed a high correlation between the quality control samples (Spearman’s r > 0.9) (Supplemental Fig. S1*B*), indicating high reliability of the output proteomics data. By searching MS data in the UniProt database, 7626 annotated proteins were identified, including a previously reported functional SEP called NoBody (35). Partial least squares discriminant analysis (PLS-DA) revealed clear separation between normal and tumor groups based on these protein profiles (Fig. 3*B*). Notably, 5700 proteins (74.7%) showed concordance with the PDC000446 dataset comprising 228 glioma tumors (Supplemental Fig. S1*C*), validating the clinical relevance of our proteomic data.

Comparative analysis revealed 1778 differentially expressed proteins between glioma tumors and normal tissues (Supplemental Fig. S1*D*, Supplemental Table S3). Gene set enrichment analysis (GSEA) of these proteins demonstrated significant upregulation of PI3K/AKT and MAPK oncogenic signaling pathways in tumors, concurrent with downregulation of p53-mediated transcriptional regulation (Supplemental Fig. S1*E*). These pathway alterations align with previous large-scale proteomic characterizations of glioma (36), confirming the robustness of our AmF-C8-SPE-derived data and the representative nature of our clinical specimens.

Subsequently, applying our stringent filtering criteria (Fig. 1*B*), we identified 549 high-confidence SEPs in glioma and normal tissues: 348 SEPs against the OpenProt database and 241 against the SmProt database, with 40 overlapping SEPs (Fig. 3*C*, Supplemental Table S4). Among 549 SEPs, 113 SEPs were dysregulated in glioma (56 upregulated, 57 downregulated) (Fig. 3*D*, Supplemental Table S5). Notably, sequence coverage analysis demonstrated robust identification quality, with 92.2% (506/549) of SEPs exhibiting >10% coverage and 19.3% (106/549) achieving >50% coverage (Fig. 3E). The coverage of our MS data is superior to the previous report (37).

Codon usage bias represents a fundamental evolutionary feature of genomes that critically influences gene expression (19, 20). Typically, AUG or near-cognate start codons (CUG, GUG, and UUG) within the Kozak consensus sequence serve as start codons (38). Analysis of translation initiation sites among 549 glioma-associated SEPs revealed that 71% utilized the canonical AUG start codon, while 19% employed near-cognate start codons (Fig. 3*F*, Supplemental Table S6). Interestingly, 18% of SEPs are initiated by other codons (Fig. 3*F*, Supplemental Table S6), consistent with previous reports that SEP translation can originate from atypical start sites (34, 39).

SEPs were reported to be derived from transcripts of pseudogenes, mRNAs, lncRNAs, and other non-coding RNAs (ncRNAs) (40, 41). Our analysis showed that 49% of glioma-associated SEPs originated from pseudogenes, with smaller proportions deriving from protein-coding genes (27%) and lncRNAs (10%) (Fig. 3*G*, Supplemental Table S6). Additionally, we calculated the instability index of SEPs using ExPASy ProtParam (42, 43) and found that the identified SEPs exhibited higher stability than annotated proteins in the Human_SwissProt database, as evidenced by their markedly lower instability index (Fig. 3*H*).

### Experimental Validation of Identified SEPs in Glioma

**Fig. 4:**
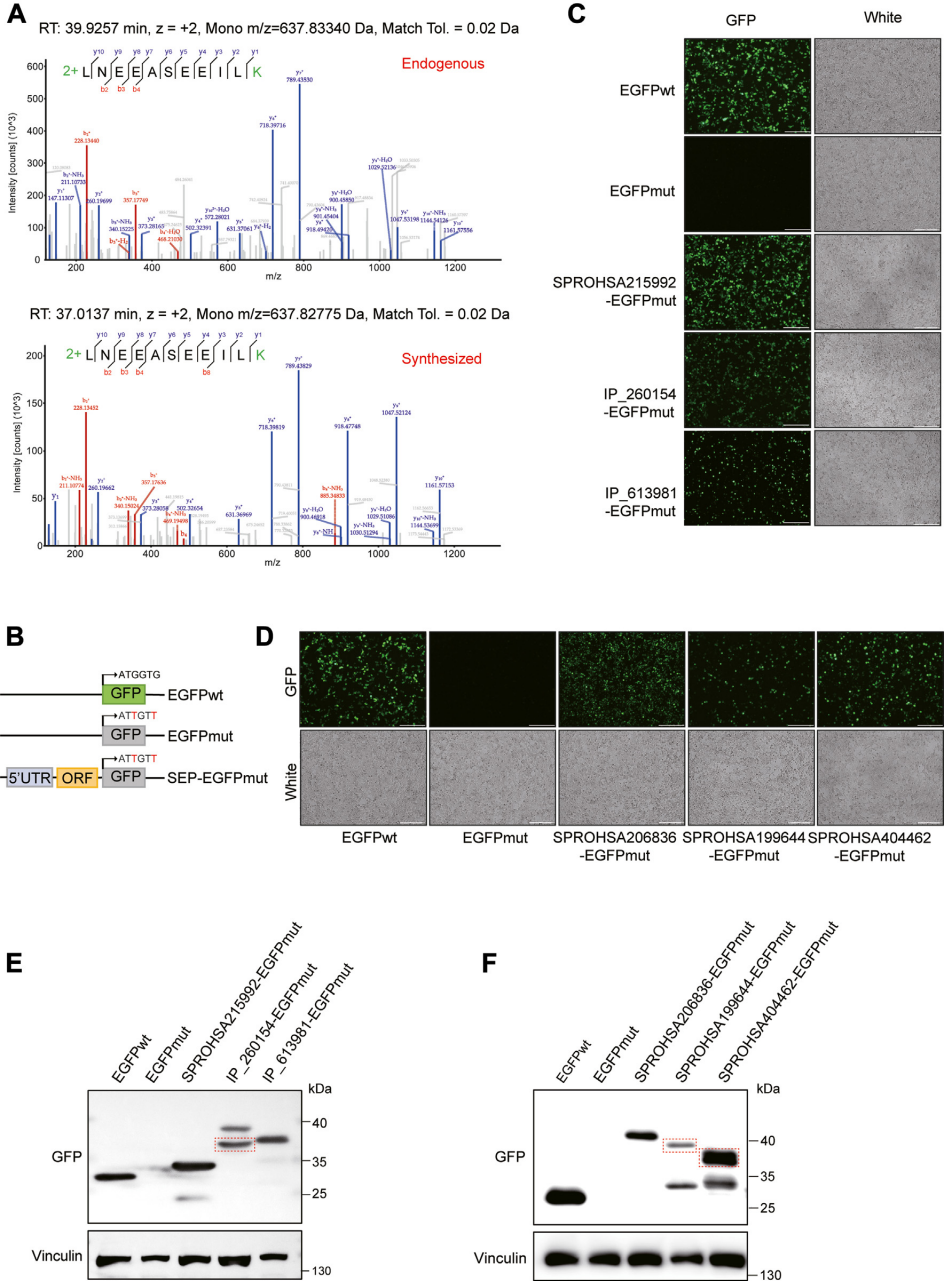
**Experimental validation of SEPs**. *A*, the MS2 spectrum of endogenous and synthesized LNEEASEEILK peptide. *B*, illustration of different constructs used in this study. *C* and *D*, detection of GFP fluorescence in HEK293T cells 48 h post-transfection with different SEP-EGFP fusion constructs. Scale bar = 250 μm. *E* and *F*, Western blot analysis of SEP-EGFP fusion proteins using anti-GFP antibody. *Red rectangles* highlight the primary SEPs. EGFP, enhanced green fluorescent protein; SEPs, small open reading frame - encoded peptides.

To validate the glioma-associated SEPs identified in this study, we employed both MS and cellular biology approaches. For MS-based validation, we synthesized peptide standards corresponding to 10 randomly selected SEPs. Comparative LC-MS/MS analyses revealed high spectral concordance between synthetic standards and endogenous peptides for eight of 10 tested SEPs (all except SPROHSA215992 and IP_613981). (Supplemental Fig. S2*A*, Supplemental Fig. S4), with representative matches such as LNEEASEEILK showing excellent spectral agreement (Fig. 4*A*). For biological validation, we developed a fusion protein expression system by cloning the 5′-UTR and ORF region of SEPs into the pEGFPmut-N1 vector, where the native EGFP start codon (ATGGTG) was mutated to ATTGTT to eliminate EGFP expression (Fig. 4*B*). We randomly selected six SEPs for validation, comprising three MS-verified candidates (SPROHSA215992, IP_260154, IP_613981) and three differentially expressed SEPs (SPROHSA206836, SPROHSA199644, SPROHSA404462; Supplemental Fig. S2*B*). Immunofluorescence microscopy imaging confirmed GFP expression exclusively in cells transfected with EGFPwt or SEP-EGFPmut constructs, but not in cells transfected with EGFPmut (Fig. 4*C*, *D*).

To further validate the expression of SEP-EGFP fusion proteins, we performed Western blot analysis using anti-GFP antibodies (Fig. 4*E*, *F*). Given that the 5′-UTR and ORF regions may contain alternative start codons, a single transcript could potentially encode multiple peptide isoforms using different translation initiation sites. As illustrated in Supplemental Figure S3, the RNA transcripts encoding IP_260154, SPROHSA199644, and SPROHSA404462 have the potential to generate alternative peptide isoforms. Consistent with this, Western blot analyses detected not only the primary SEPs but also their corresponding alternative isoforms (Fig. 4*E*, *F*). Together, these results provide compelling experimental evidence supporting the existence of glioma-associated SEPs and their alternatively translated variants.

### Comprehensive Analysis of SEPs Implicated in Glioma Progression

**Fig. 5:**
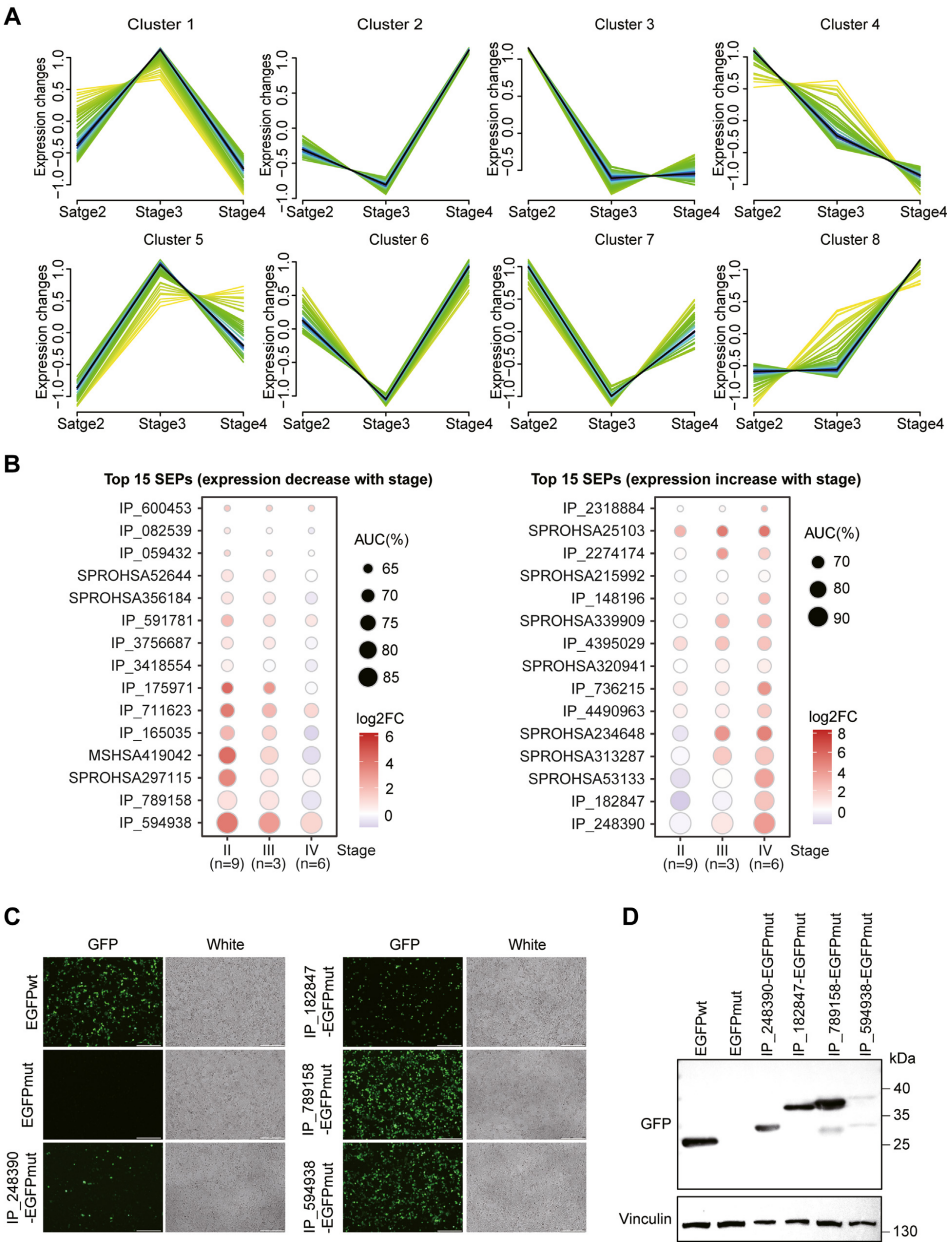
**Identification of SEPs associated with glioma grade.***A*, Mfuzz clustering analyses of SEP expression in glioma. *B*, the dot plot revealed that the top 15 SEPs with significant grade-dependent decreases (*left*) or increases (*right*) in expression. *C*, detection of GFP fluorescence in HEK293T cells for 48 h post-transfection with candidate SEP-EGFP fusion constructs. Scale bar = 250 μm. *D*, Western blot analysis of candidate SEP-EGFP fusion proteins using anti-GFP antibody. AUC, area under the curve; EGFP, enhanced green fluorescent protein; SEPs, small open reading frame - encoded peptides.

SEPs exhibiting glioma grade-dependent expression patterns represent promising candidates for diagnostic biomarkers and therapeutic targets. Using Mfuzz clustering analysis, we identified eight distinct SEP clusters showing different SEP expression patterns that correlate with glioma progression stages (Fig. 5*A*). Notably, 64 SEPs displayed progressive downregulation with increasing tumor grade (clusters 3 and 4), while 49 showed reciprocal upregulation (clusters 5 and 8). The glioma grade-dependent expression changes of these 113 SEPs were confirmed by a ROC curve analysis and calculation of their corresponding AUC values (Supplemental Table S7). Furthermore, the top 15 SEPs with high AUC values indicating significantly decreased or increased expression changes are presented in Figure 5*B*. Among them, IP_789158 and IP_594938 showed the most pronounced negative correlation with glioma progression, whereas IP_182847 and IP_248390 exhibited the strongest positive association, suggesting their potential functional significance during glioma development (Fig. 5*B*, Supplemental Table S7). To confirm the expression of these four SEPs, we cloned their 5′-UTR and ORF regions into the pEGFPmut-N1 plasmid to generate GFP fusion constructs as shown in Figure 4*B*. Both immunofluorescence imaging and Western blot analyses revealed that both EGFPwt and SEP-EGFPmut constructs successfully expressed GFP, while no GFP expression was detected in cells transfected with EGFPmut (Fig. 5*C*, *D*), supporting the reliability of our identified SEPs. Therefore, these findings highlight the clinical potential of grade-associated SEPs as diagnostic biomarkers and therapeutic targets for glioma management.

### Subcellular Location of the SEPs Detected in Glioma

**Fig. 6:**
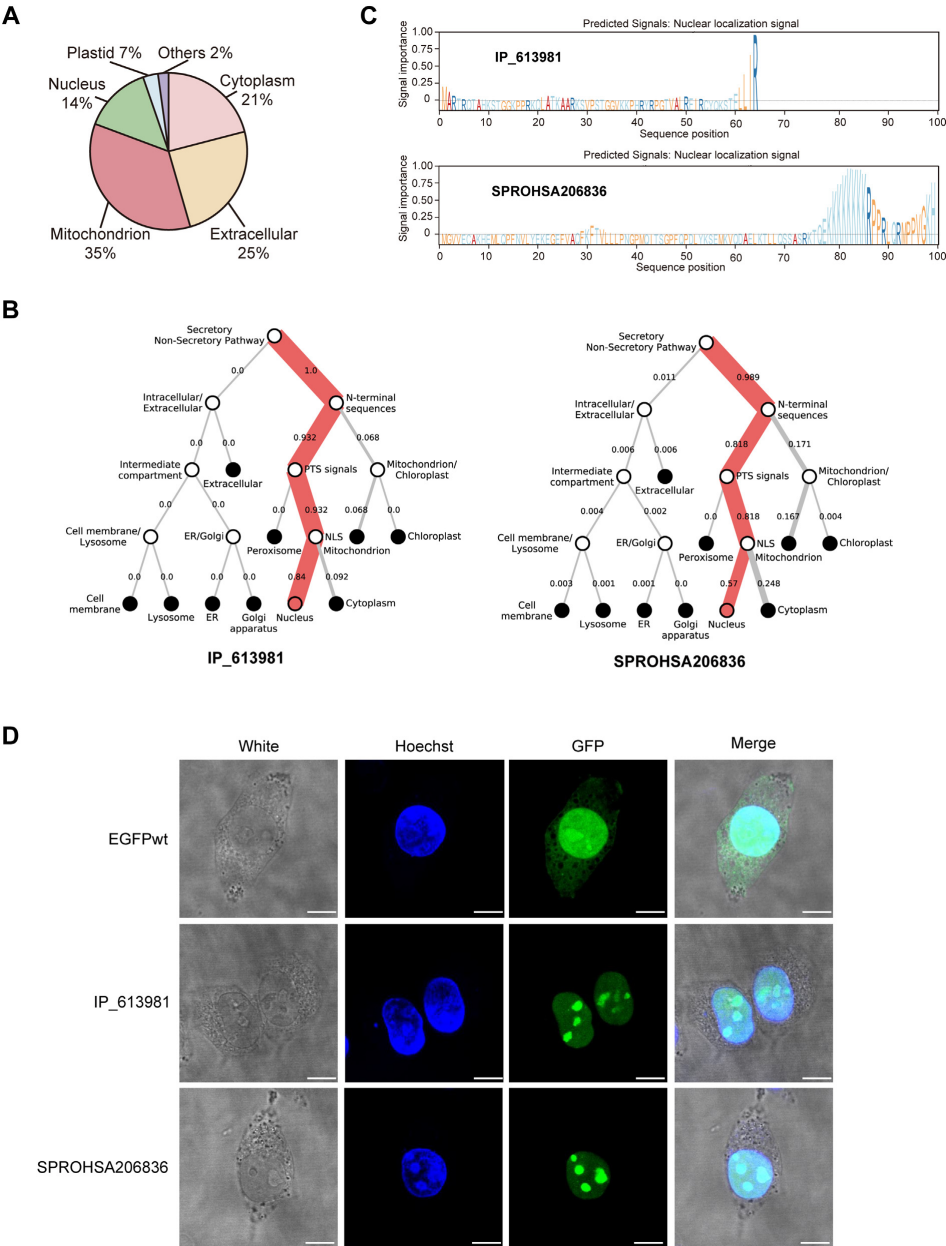
**Subcellular localization identification of SEPs**. *A*, DeepLoc predicts subcellular localization of identified SEPs. *B*, Hierarchical tree and (*C*) Sorting signal of SPROHSA206836 and IP_613981. *D*, confocal microscopy of HEK293T cells 48h post-transfection showing GFP fluorescence (*green*), Hoechst-stained nuclei (*blue*), and brightfield morphology. Scale bar = 10 μm. GFP, green fluorescent protein; SEPs, small open reading frame - encoded peptides.

Subcellular localization of SEPs provides critical insights into SEP function. Using DeepLoc (44, 45), we predicted the cellular distribution of all 549 glioma-associated SEPs based on characteristic features including signal peptides, transmembrane domains, and conserved motifs. Our analysis revealed distinct compartmentalization patterns, with predominant mitochondrial localization (35%), followed by extracellular (25%), cytoplasmic (21%), and nuclear distributions (14%) (Fig. 6*A*, Supplemental Table S6). For the experimentally validated SEPs shown in Figure 4, DeepLoc predicted the nuclear localization for both IP_613981 and SPROHSA206836, identifying their characteristic nuclear localization signals (NLS): TELLIR in IP_613981 and TQEKKKKKKKKRPPRLQRMPPVGKH in SPROHSA206836 (Fig. 6, *B* and *C*). To confirm the predictions, we transfected SEP-GFPmut plasmids into cells and performed confocal microscopy. The results showed that both SEPs from IP_613981 and SPROHSA206836 proteins were mainly localized in the nucleus (Fig. 6*D*). These results only map the subcellular distribution of glioma-associated SEPs, but also establish a foundation for investigating their functional roles.

## Discussion

Recent advancements in MS technology have promoted SEP identification. However, due to SEP’s low abundance and small molecular size, MS-based SPE identification remains challenging. To overcome these limitations, we developed an effective small peptide-enrichment strategy combining traditional C8 SPE with ammonium formate (AmF) buffer and gradient elution (AmF-C8-SPE). This optimized approach demonstrated superior performance compared to the Classic-C8 SPE method, yielding both greater SEP identification number and higher-quality spectra (Fig. 1). Applying this advanced workflow to clinical glioma specimens, we identified 549 novel SEPs in paired tumor/normal tissues. To the best of our knowledge, this is the first study to comprehensively characterize SEPs in human glioma samples.

C8 SPE has emerged as a fundamental technique for peptide enrichment, utilizing hydrophobic C8-modified silica sorbents to selectively isolate peptide hormones from complex biological samples (46, 47). Since its adaptation for SEP enrichment by Ma *et al* (19), where it outperformed both acid precipitation and 30-kDa MWCO filtration, C8 SPE has been widely employed for SEP discovery across various biological samples (20, 21). The conventional Classic-C8 method uses TEAF buffer to enhance SEP adsorption but requires subsequent methanol/chloroform precipitation to remove MS-incompatible TEAF, potentially leading to substantial loss of hydrophilic proteins (20, 48). To address these limitations, we developed an optimized AmF-C8-SPE protocol featuring two key modifications: (1) substitution of TEAF buffer with MS-compatible AmF buffer, thereby eliminating precipitation requirements, and (2) implementation of gradient elution with ACN/AmF buffer to improve fractionation efficiency. This advanced workflow demonstrated significant improvements over Classic-C8, achieving higher unique peptide identification rates, enhanced sequence coverage, and a 3-fold increase in SEP detection (Fig. 2*G*-*I*). To our knowledge, this method represents the most effective single-step SEP enrichment protocol reported to date.

The selection of enrichment methodology can significantly influence SEP identification outcomes, as different techniques exhibit distinct biases in microprotein recovery. For instance, Zhang *et al*. showed minimal overlap (7.58%) between SPEs identified using C8-SPE and 30-kDa MWCO filtration, while their combination enabled detection of 762 novel SEPs across diverse tissues and cell lines (20). Method performance also varies by sample type. For example, Ma *et al*. showed that C8-SPE outperformed both 30-kDa MWCO filtration and acid precipitation in K562 cells (19), whereas Yang *et al*. observed no such advantages in mouse liver tissue (17). These findings suggest that a multi-method approach may be necessary for comprehensive SEP discovery. Although our current work optimized the AmF-C8-SPE approach, future studies should investigate its synergistic combination with other methods (e.g., SEC, 30-kDa MWCO filtration, or solvent precipitation) to achieve more SEP identification.

Accumulating evidence indicates that SEP-encoding transcripts often harbor multiple potential start codons, including canonical AUG and near-cognate start codons, enabling the production of distinct SEP isoforms via alternative translation initiation. For instance, the lncRNA TUNAR encodes not only the 48-amino acid microprotein pTUNAR but also a 65-amino acid isoform translated from an upstream in-frame AUG codon (49). Similarly, Na *et al*. reported that translation initiation at a non-canonical AGG codon within an upstream open reading frame (uORF) of *LAMA3* mRNA produces the alt-LAMA3 isoform (39). Consistent with these findings, our study identified multiple SEP isoforms derived from transcripts encoding IP_260154, SPROHSA196644, and SPROHSA196645 (Fig. 4*E*, *F*). Given their sequence divergence, these isoforms may participate in different biological pathways, underscoring the necessity for further functional investigation.

Beyond alternative translation, post-translational modifications (PTMs) of SEPs likely contribute to their functional diversification. Currently, no studies have characterized SEP-specific PTMs, highlighting an important area for further research. Compelling evidence demonstrates the importance of subcellular localization in determining SEP function. For example, the mitochondria-localized SEP ASAP (encoded by LINC00467) regulates mitochondrial respiration and ATP synthesis (50), while the nuclear-localized alt-LAMA3 isoform participates in pre-rRNA transcription (39). Our localization studies revealed that IP_613981 and SPROHSA206836 accumulate in the nucleus (Fig. 2*D*), suggesting potential roles in nucleolar architecture or chromatin dynamics. To interrogate these hypotheses, CRISPR-Cas9-mediated knockout or knockin approaches could be employed to dissect the underlying mechanisms.

Emerging evidence demonstrates that SEPs may play oncogenic or tumor-suppressive roles in glioma progression (51). For example, the 404-amino-acid MET variant (MET404) encoded by circMET promotes glioblastoma invasiveness through interaction with the β subunit of MET kinase to form a constitutively activated MET receptor complex (52). In contrast, MP31, a mitochondrial-localized microprotein encoded by the upstream open reading frame (uORF) of the PTEN gene, has been shown to disrupt mitochondrial quality control and inhibit glioblastoma tumorigenesis (53). Despite these advances, the molecular mechanisms underlying SEP functions remain largely unexplored. In the future, we will employ gain and loss-of-function strategies to investigate their functions *in vitro* and *in vivo* and elucidate SEP-mediated regulation of key signaling pathways in glioma progression.

In summary, we developed an optimized SEP enrichment method coupled with advanced MS to identify 549 novel SEPs in glioma tissues, including 113 with differentially expressed between tumors and normal tissues. This study expands our understanding of non-coding element annotation in the genome and provides new perspectives for screening novel glioma biomarkers and therapeutic targets.

## Data Availability

The mass spectrometry raw data and research results have been deposited to the in the ProteomeXchange Consortium (http://proteomecentral.proteomexchange.org) via the iProX (54) with the dataset identifier of PXD058073.

## Supplemental Data

This article contains supplemental data.

## Conflict of Interest

The authors declare that they have no conflicts of interest with the contents of this article.
