# Supplementary material for: Identification of Small Open Reading Frame-Encoded Peptides in Glioma by an Optimized Proteomics Strategy
Source: Mol Cell Proteomics. 2025 Jun 11;24(7):101016. doi: 10.1016/j.mcpro.2025.101016 (PMC12275937; doi:10.1016/j.mcpro.2025.101016)
Supplement: Supplemental Figures [file mmc1.pdf]

Supplementary information

Identification of Small Open Reading Frame-encoded Peptides in Glioma by an Optimized Proteomics Strategy

Tingting Zhang[1‡], Jian Cheng[2‡], Jiao Li[1], Zixia Ye[1], Na Li[3], Jifeng Wang[3], Xiaojuan Yang[1*], Yong Peng[1*]

[1] Center for Molecular Oncology, Frontiers Science Center for Disease-related Molecular Network, State Key Laboratory of Biotherapy and Cancer Center, West China Hospital, Sichuan University, Chengdu 610041, China.

[2] Department of Neurosurgery, West China Hospital, Sichuan University, Chengdu 610041, China.

[3] Laboratory of Protein and Peptide Pharmaceuticals & Laboratory of Proteomics, Institute of Biophysics, Chinese Academy of Sciences, Beijing 100101, China

[‡] These authors contributed equally to this work.

[*] Correspondence authors (Xiaojuan Yang, e-mail: yangxiaojuan@wchscu.edu.cn; Yong Peng, e-mail: yongpeng@scu.edu.cn.

Running title: Identification of Small Open Reading Frame-encoded Peptides in Glioma

This file includes:

Supplementary Fig. S1

Supplementary Fig. S2

Supplementary Fig. S3

Supplementary Fig. S4

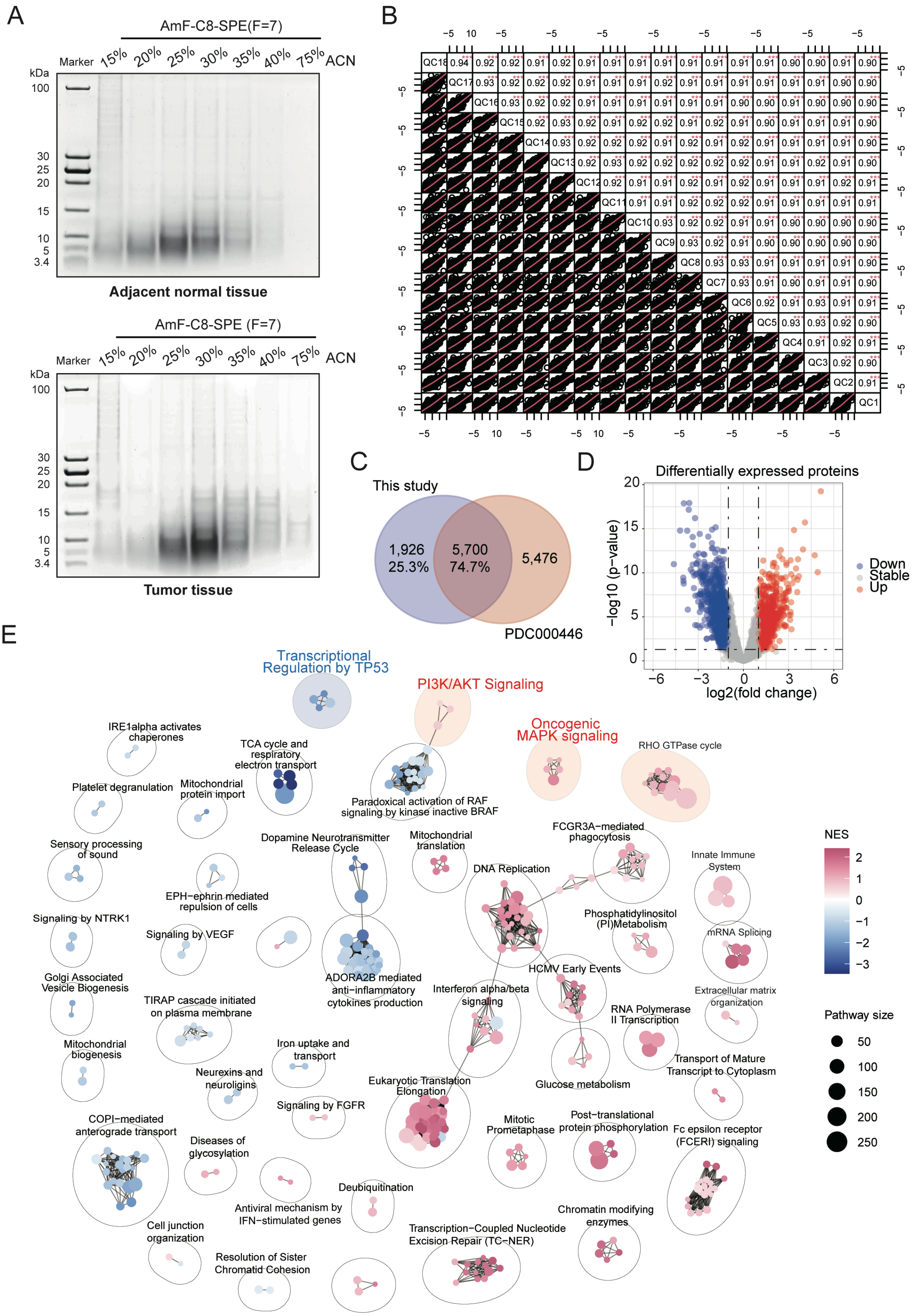

A
AmF-C8-SPE(F=7)
Marker 15% 20% 25% 30% 35% 40% 75% ACN
kDa
100
30
25
20
15
10
5
3.4
Adjacent normal tissue
AmF-C8-SPE (F=7)
Marker 15% 20% 25% 30% 35% 40% 75% ACN
kDa
100
30
25
20
15
10
5
3.4
Tumor tissue
B
QC18 0.94 0.92 0.92 0.91 0.92 0.91 0.91 0.91 0.91 0.91 0.91 0.91 0.91 0.90 0.90 0.91 0.90
QC17 0.93 0.92 0.92 0.92 0.91 0.91 0.91 0.92 0.91 0.91 0.91 0.91 0.91 0.91 0.90 0.90
QC16 0.93 0.92 0.92 0.91 0.91 0.91 0.91 0.91 0.91 0.90 0.90 0.91 0.90 0.90 0.90
QC15 0.92 0.93 0.92 0.91 0.92 0.92 0.91 0.92 0.91 0.90 0.91 0.91 0.91 0.90
QC14 0.93 0.92 0.92 0.92 0.91 0.92 0.91 0.92 0.91 0.91 0.91 0.91 0.90
QC13 0.92 0.93 0.92 0.92 0.92 0.92 0.92 0.91 0.91 0.92 0.91 0.90
QC12 0.92 0.91 0.91 0.92 0.91 0.92 0.91 0.91 0.91 0.92 0.91
QC11 0.91 0.91 0.92 0.92 0.92 0.91 0.91 0.91 0.91 0.91
QC10 0.93 0.92 0.92 0.91 0.91 0.90 0.90 0.90 0.90
QC9 0.93 0.92 0.91 0.90 0.90 0.90 0.90 0.90
QC8 0.93 0.93 0.91 0.91 0.91 0.91 0.90
QC7 0.93 0.91 0.90 0.91 0.91 0.90
QC6 0.92 0.91 0.93 0.91 0.91
QC5 0.93 0.93 0.92 0.90
QC4 0.91 0.92 0.91
QC3 0.92 0.90
QC2 0.91
QC1
C
This study
1,926
25.3%
5,700
74.7%
5,476
PDC000446
D
Differentially expressed proteins
−log10 (p−value)
log2(fold change)
Down
Stable
Up
E
Transcriptional Regulation by TP53
PI3K/AKT Signaling
Oncogenic MAPK signaling
RHO GTPase cycle
IRE1alpha activates chaperones
TCA cycle and respiratory electron transport
Platelet degranulation
Mitochondrial protein import
Paradoxical activation of RAF signaling by kinase inactive BRAF
Sensory processing of sound
Dopamine Neurotransmitter Release Cycle
Mitochondrial translation
FCGR3A−mediated phagocytosis
DNA Replication
Innate Immune System
EPH−ephrin mediated repulsion of cells
Signaling by NTRK1
Signaling by VEGF
Phosphatidylinositol (PI)Metabolism
mRNA Splicing
ADORA2B mediated anti−inflammatory cytokines production
HCMV Early Events
Golgi Associated Vesicle Biogenesis
Interferon alpha/beta signaling
Extracellular matrix organization
TIRAP cascade initiated on plasma membrane
RNA Polymerase II Transcription
Mitochondrial biogenesis
Iron uptake and transport
Transport of Mature Transcript to Cytoplasm
Neurexins and neuroligins
Eukaryotic Translation Elongation
Glucose metabolism
COPI−mediated anterograde transport
Signaling by FGFR
Mitotic Prometaphase
Post−translational protein phosphorylation
Fc epsilon receptor (FCERI) signaling
Diseases of glycosylation
Deubiquitination
Antiviral mechanism by IFN−stimulated genes
Chromatin modifying enzymes
Cell junction organization
Transcription−Coupled Nucleotide Excision Repair (TC−NER)
Resolution of Sister Chromatid Cohesion
NES
Pathway size
50
100
150
200
250

Supplementary Fig. S1 Bioinformatics analysis of annotated proteins in glioma. (A) Tricine SDS-PAGE analysis of SEPs enriched by AmF-C8-SPE (fractions = 7) from glioma tumor and adjacent normal tissue. (B) Spearman's correlation of the quantify control. (C) Venn diagram of annotated proteins identified in this study and database data. (D) Volcano plot of annotated proteins in glioma tumor and adjacent normal tissue. (E) Gene Set Enrichment Analysis (GSEA) of differentially expressed proteins between glioma tumor and adjacent normal tissue. AmF-C8-SPE, ammonium formate-mediated C8 solid-phase enrichment; F, fractions.

A

SEP names	Synthesized peptides	AAs	MW [kDa]	Fold change	p-value	Identified times
SPROHSA111613	YLYTLVISEK	38	4.5	0.870	0.582	36
SPROHSA149972	QANLYISEGLHPR	36	4.3	1.4190	0.215	34
SPROHSA177413	TSQGAEEVSDVEGGVEK	82	8.2	0.960	0.900	31
SPROHSA215992	GPAVGIDLGTTYSCVGVFHQGK	35	3.6	0.776	0.037	36
SPROHSA262723	HVFGESDKLIGQK	68	7.5	1.077	0.603	36
IP_260154	TSQGAEEVSDVEGGVEK	82	8.2	1.102	0.763	30
IP_613981	KSVPSTGGVKKPHR	64	7.2	1.507	0.255	35
IP_603079	LNEEASEEILK	225	25.9	0.775	0.467	34
IP_620044	QANLYISEGLHPR	65	7.4	1.601	0.097	34
IP_767719	ISTHLVIR	48	5.8	0.690	0.009	35

B

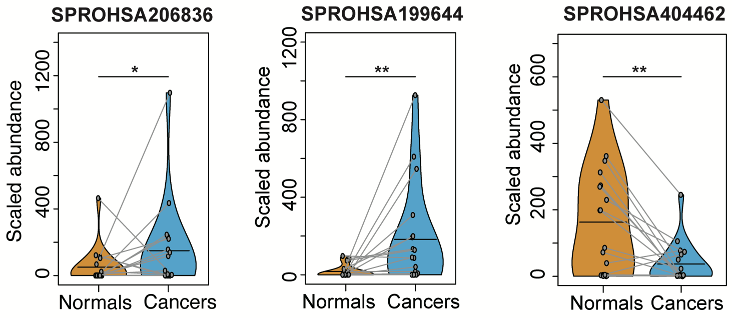

Supplementary Fig. S2 Experimental Validation of Identified SEPs. (A) 10 unique peptides from randomly selected 10 SEPs were synthesized as peptide standards. (B) Violin plots showed the expression of SPROHSA206836, SPROHSA199644 and SPROHSA404462 in glioma tumors and normal tissues. SEP, small open reading frame-encoded peptide. AAs, amino acids; MW, molecular weight.

A

GAGTTGCCAGTAACGGACATGGCTGCGGCCCCCGGAGGAGGGGACGTGAAGTGAGGAGGGGG ENST000005810
TTGGGAGGGGAGAGGACGCGGGCGAGGAAGACCAGCCCCGGGGCCCCG ATG TTG TCA CTG ENST000005810
M L S L IP_260154-isoform
TCA CAG ACT CAC TGG GGT TTG TAC ATG CTG GGG AGG AGC CTT CCT TTC AGG GGT GAC CAC ENST000005810
S Q T H W G L Y M L G R S L P F R G D H IP_260154-isoform
M L G R S L P F R G D H IP_260154
ATT CAT CTG GGC ATG CCT GCA GTA CTC TTG GCC CAT GGA CCT GAA GGA GAA GCA CCT GGG ENST000005810
I H L G M P A V L L A H G P E G E A P G IP_260154-isoform
I H L G M P A V L L A H G P E G E A P G IP_260154
CGA GCC TCC CTC AGC CCT GGG CCT GTC CAC GCG GAA GGC CCT CAG CGT CCT GAA GGA GCA ENST000005810
R A S L S P G P V H A E G P Q R P E G A IP_260154-isoform
R A S L S P G P V H A E G P Q R P E G A IP_260154
GCT GGA GGC AGT GCT GGA AGG ACA TCT CAG GGA GCG GAA GAA GTG TCT GAC GTG GAA GGA ENST000005810
A G G S A G R T S Q G A E E V S D V E G IP_260154-isoform
A G G S A G R T S Q G A E E V S D V E G IP_260154
GGT GTG GAG AAG CAG CTT CCT CCA CCA CAG TAA TAACCGCTGCTCCTGCTTCCACTGGCCGGGGGCCTC ENST000005810
G V E K Q L P P P Q * IP_260154-isoform
G V E K Q L P P P Q * IP_260154
ACTCATGCTACTGGCCGT……………………………AAACCCCATCATTACCAAGCTGTACCAGAG ENST000005810

B

ATG GCT GGG CTG CCC AGC AGG ATC GTC AAG GAA ACC CAG CTT TTG CTG GCA GAA CCA GTT ENST00000513791.1
M A G L P S R I V K E T Q L L L A E P V SPROHSA199644
CCT GGC CTC AAA GCA GAA CCA TAT GAG AGC AAC ATC CAT TAT TTT CAT GTG GTC ATT GCC ENST00000513791.1
P G L K A E P Y E S N I H Y F H V V I A SPROHSA199644
AGT CCC TGG GAT TCC CAC TGT GAG GGA GGG ACT TTT AAA CTT GGA CTA TTT CTT CCA GAA ENST00000513791.1
S P W D S H C E G G T F K L G L F L P E SPROHSA199644
GAA TAC CCA ATG GCA GCC CCT AAA GTG CGT TTT ATG ACC AAA ATT TGT CAT CCT AAT GTA ENST00000513791.1
E Y P M A A P K V R F M T K I C H P N V SPROHSA199644
M T K I C H P N V SPROHSA199644-isoform
GAC AAG TTG GGG AGA ATA CGT TAA GATATTTTGAAAGGTAAGTGGTCCCCAGCCCTGCAGATCCGCACAGTTCT ENST00000513791.1
D K L G R I R * SPROHSA199644
D K L G R I R * SPROHSA199644-isoform
GCTACGGATCCAGGCCTT……………………………ACTAGGCTATATGCCATGAATAATATTTAA ENST00000513791.1

C

TGCCGCTGCCATAGCCCAGCAGTTCGTGCAGGCCCACCTGTACATCGAAGGAGGGCCCCATC ENST00000416030.1
AGGACGATGTACAAGTCCTTGTCACTCTCCTCCAGAACGGTGAGCTTCTCCCACTGCGTG
GCTTAGACCACAGCCAGGACGTTCCCCAGTGACACGTTCTTAAAGCCTTCCGTCTGCTTCAGG
TCATTGTAGTTGGAG ATG TTG ATG CCG GCA GCG ATG CCA GAG CCA GCG AAG GTA AGA ACA ENST00000416030.1
M L M P A A M P E P A K V R T SPROHSA404462
TCC ACG GAG GTG AAG TCA GGG GTG AGG AAC TTG TCT TTC TCA AAG GCT GGG GAC CAG GGC ENST00000416030.1
S T E V K S G V R N L S F S K A G D Q G SPROHSA404462
AAC TCC TTC AGC AGC TGC TCT GCG CTC ACC ACC AGC CAC TCA AAC TTG GCA CTC ATG GCC ENST00000416030.1
N S F S S C S A L T T S H S N L A L M A SPROHSA404462
M A SPROHSA404462-isoform
TTG TTC ACC ACG GCT ACG AAG CCT TCA CAT ACT CCT CGG GAA CCA AAG GAG TCA CGG TAG ENST00000416030.1
L F T T A T K P S H T P R E P K E S R * SPROHSA404462
L F T T A T K P S H T P R E P K E S R * SPROHSA404462-isoform
CTCTGGATGAACTCGATG……………………………TGGTACAATTACCAAAGAAATAGGTGGTG ENST00000416030.1

Supplementary Fig. S3 RNA sequences and their predicted amino acid sequences. (A) ENST000005810 encodes IP_260154 (8.2 kDa) and its peptide isoform (9.7 kDa). (B) ENST00000513791.1 encodes SPROHSA199644 (9.8 kDa) and its peptide isoform (1.9 kDa). (C) ENST00000416030.1 encodes SPROHSA404462 (7.9 kDa) and its peptide isoform (2.4 kDa).

SPROHSA111613_ YLYTLVISEK

PYZ-1_4_20221003170221.raw #51782 RT: 49.3397 min
FTMS, 614.8463@hcd30.00, z=+2, Mono m/z=614.84571 Da, MH+=1228.68414 Da, Match Tol.=0.02 D

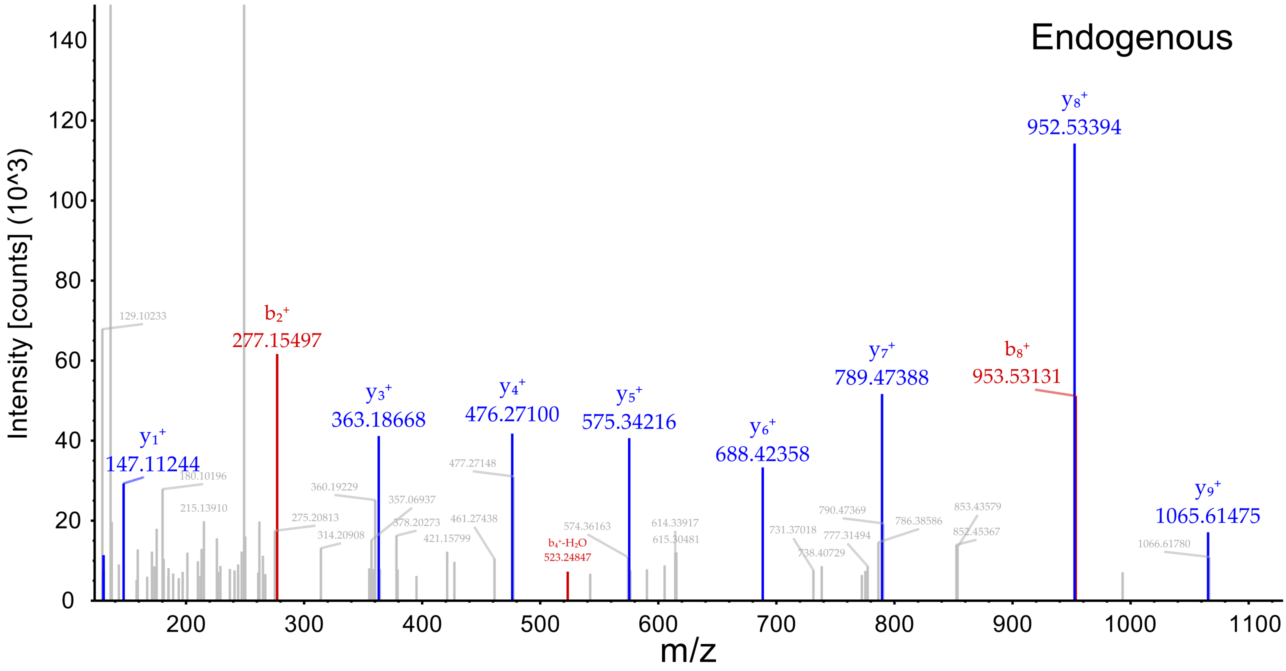

ZTT-3.raw #30071 RT: 52.4820 min
FTMS, 614.3449@hcd30.00, z=+2, Mono m/z=614.84494 Da, MH+=1228.68260 Da, Match Tol.=0.02 D

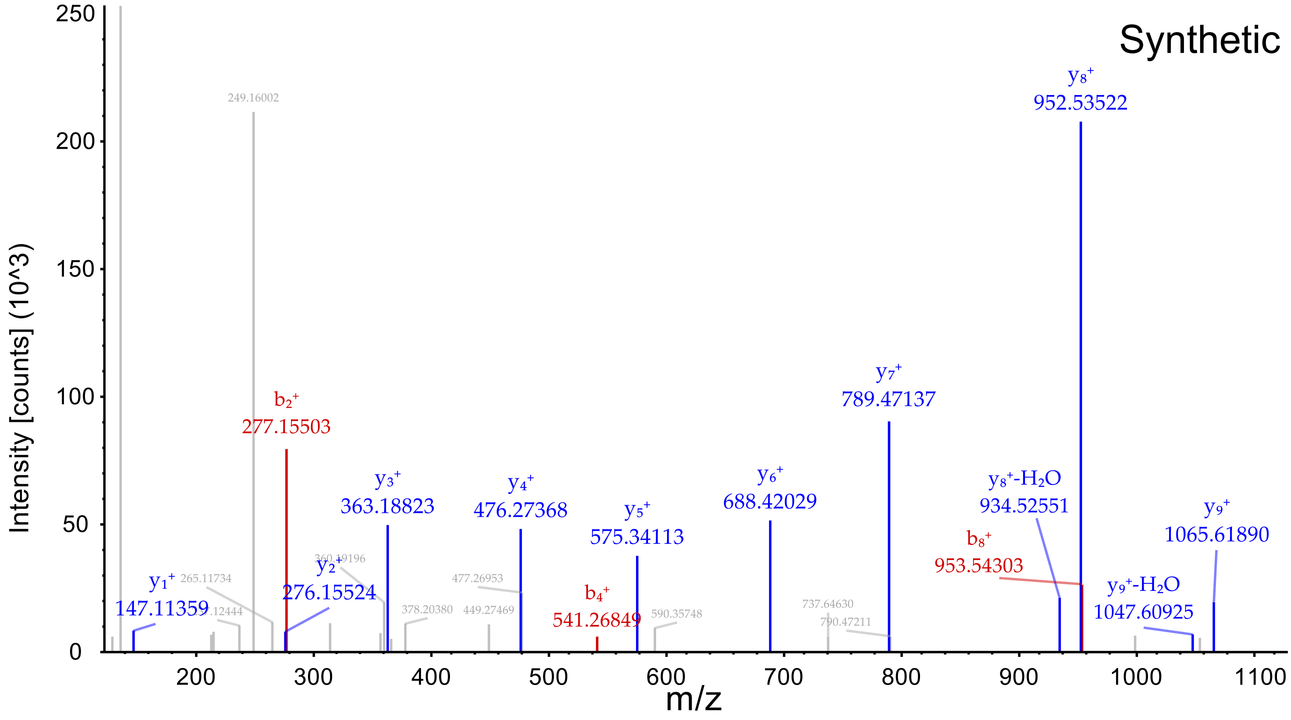

SPROHSA149972_ QANLYISEGLHPR

PYZ-2_6.raw #53786 RT: 45.4021 min
FTMS, 749.3935@hcd30.00, z=+2, Mono m/z=749.39226 Da, MH+=1497.77723 Da, Match Tol.=0.02 D

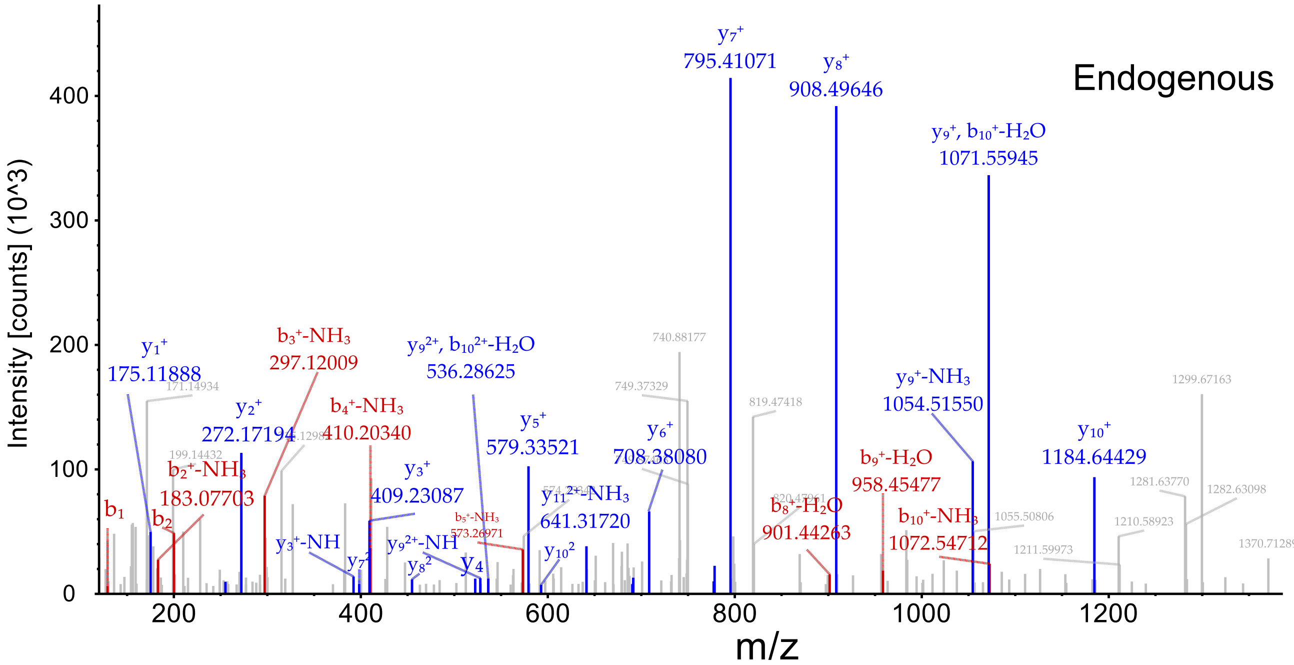

ZTT-2.raw #27056 RT: 45.1415 min
FTMS, 499.9326@hcd30.00, z=+3, Mono m/z=499.93200 Da, MH+=1497.78146 Da, Match Tol.=0.02 D

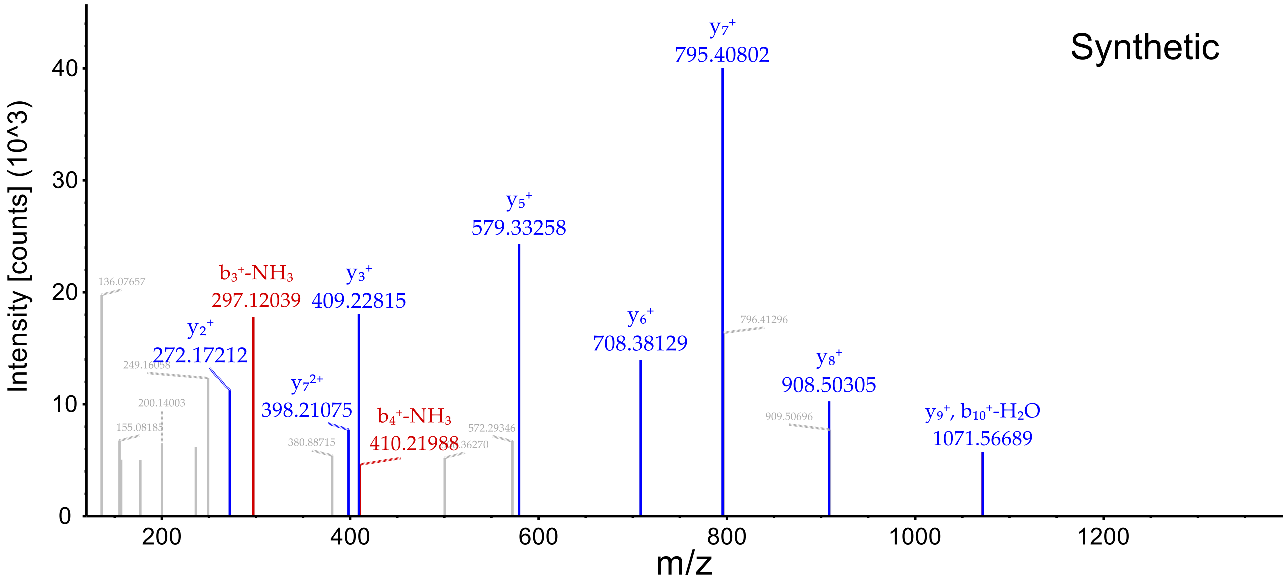

SPROHSA177413_TSQGAEEVSDVEGGVEK

PYZ-2_2.raw #41380 RT: 36.6434 min
FTMS, 860.8981@hcd30.00, z=+2, Mono m/z=860.89682 Da, MH+=1720.78635 Da, Match Tol.=0.02 D

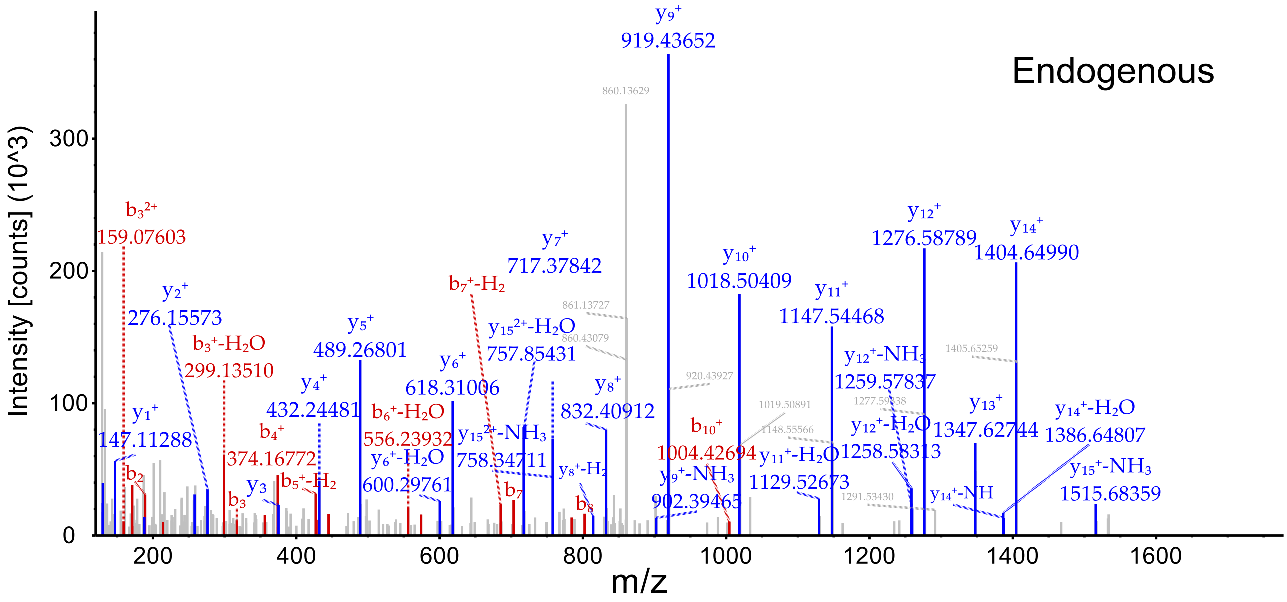

ZTT-1.raw #24872 RT: 35.0387 min
FTMS, 861.3545@hcd30.00, z=+2, Mono m/z=860.89690 Da, MH+=1720.78653 Da, Match Tol.=0.02 D

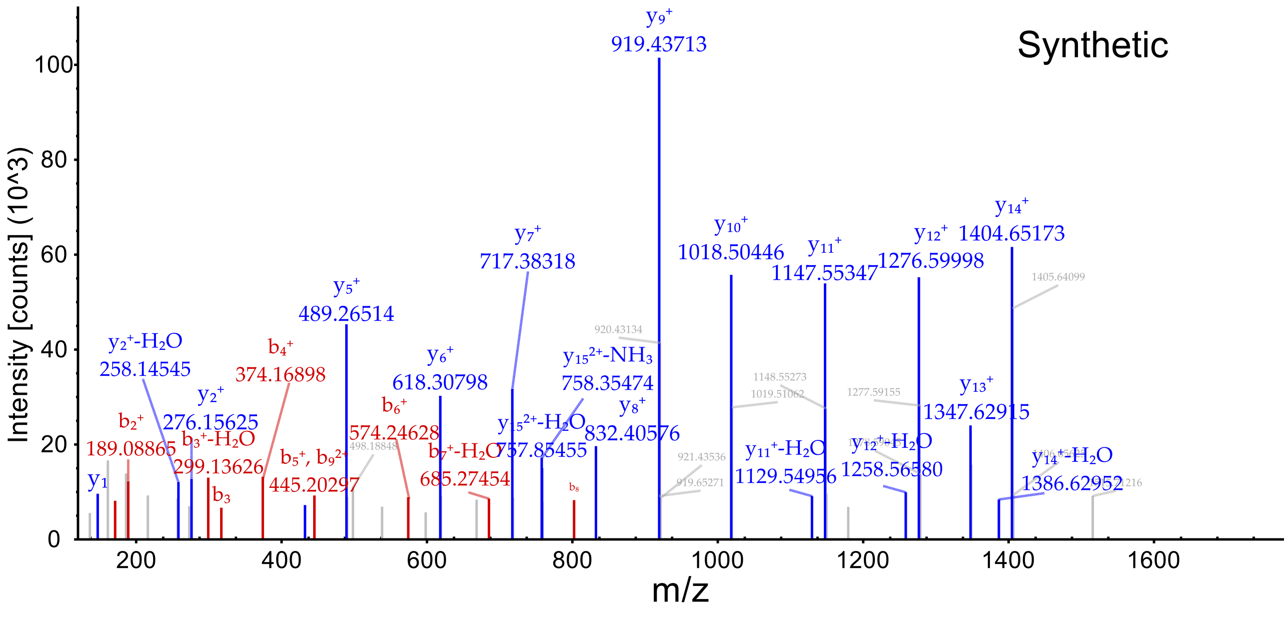

SPROHSA215992_GPAVGIDLGTTYSCVGVFHQGK

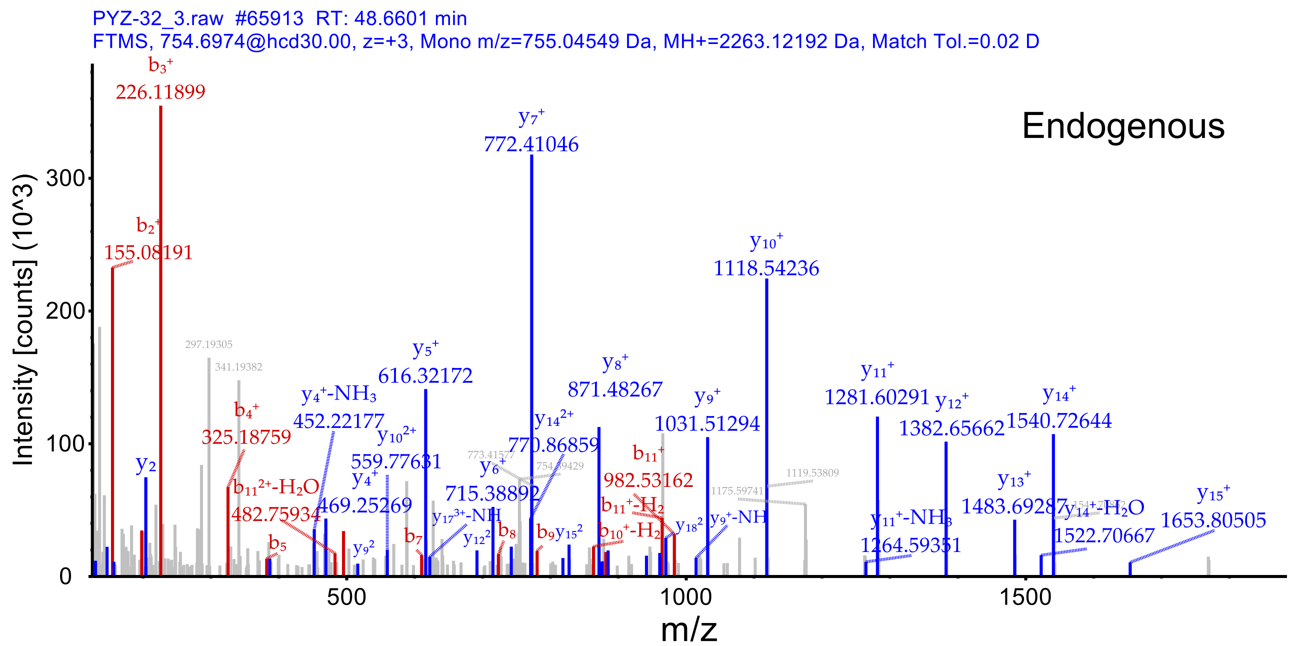

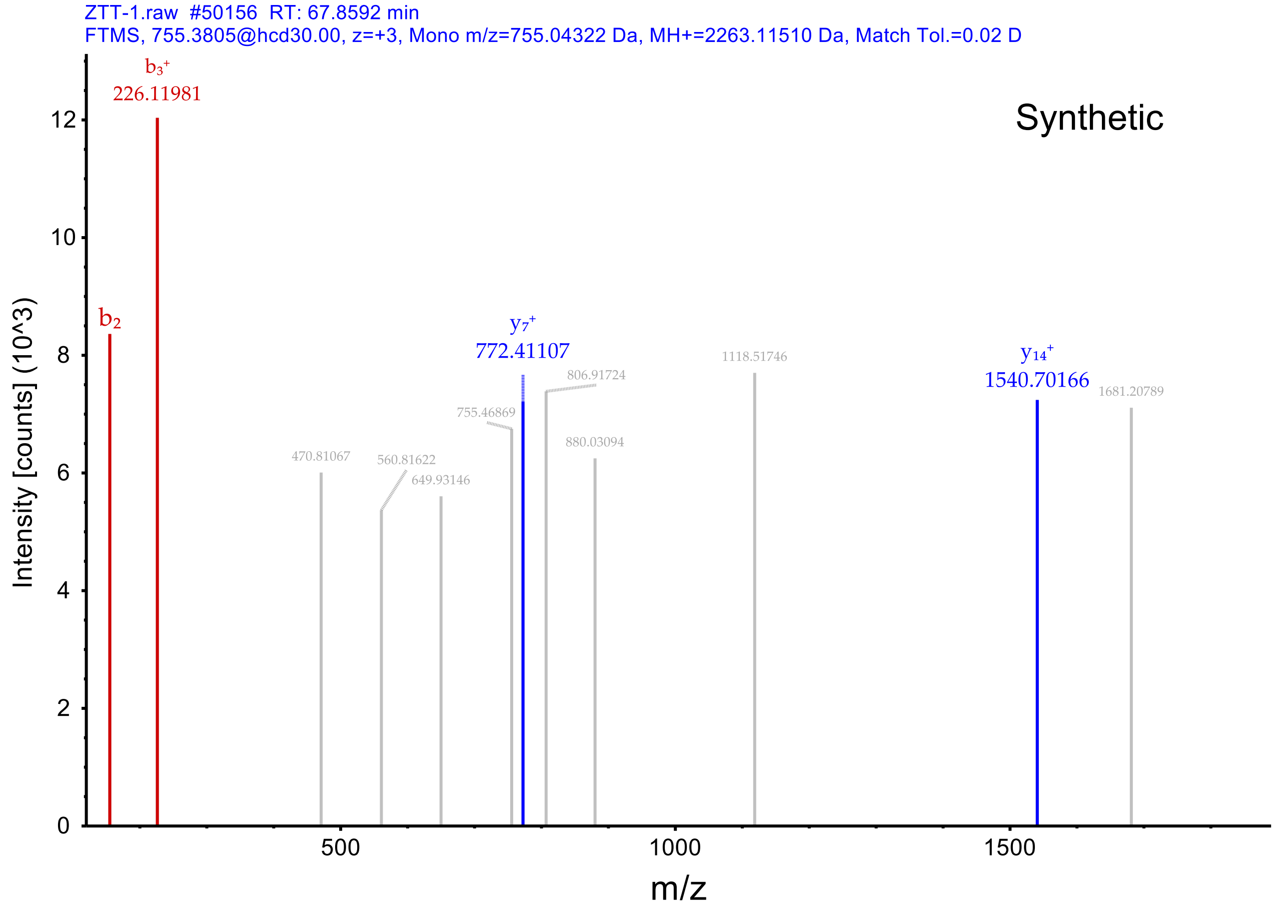

SPROHSA262723_ HVFGESDKLIGQK

PYZ-46_7.raw #40257 RT: 43.4560 min
FTMS, 487.2650@hcd30.00, z=+3, Mono m/z=486.59713 Da, MH+=1457.77682 Da, Match Tol.=0.02 D

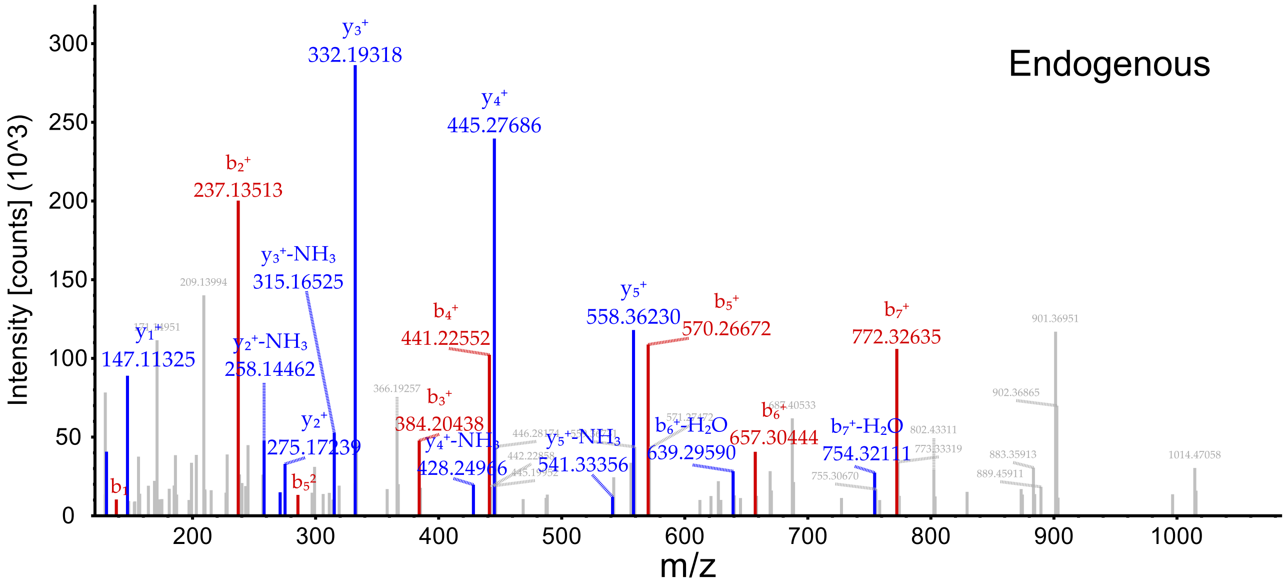

ZTT-1.raw #30120 RT: 41.6433 min
FTMS, 486.5970@hcd30.00, z=+3, Mono m/z=486.59605 Da, MH+=1457.77361 Da, Match Tol.=0.02 D

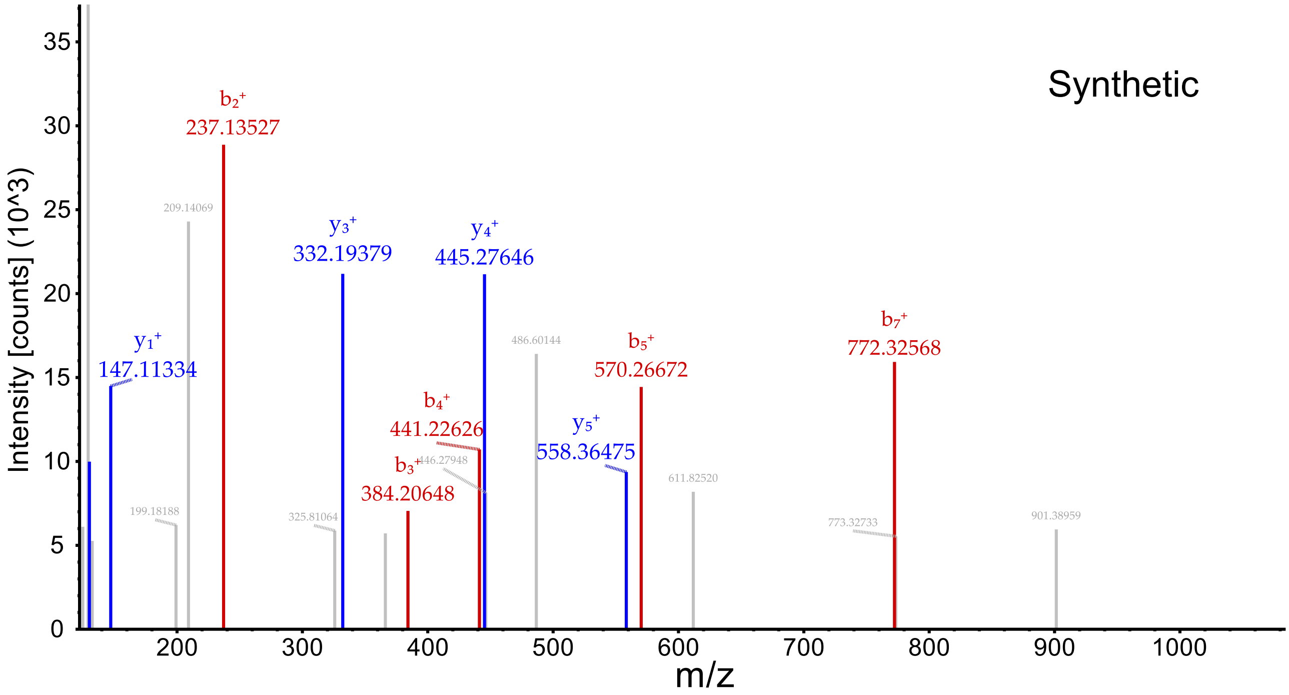

IP_260154_ TSQGAEEVSDVEGGVEK

PYZ-2_2.raw #41380 RT: 36.6600 min
FTMS, 860.8981@hcd30.00, z=+2, Mono m/z=860.89674 Da, MH+=1720.78621 Da, Match Tol.=0.02 D

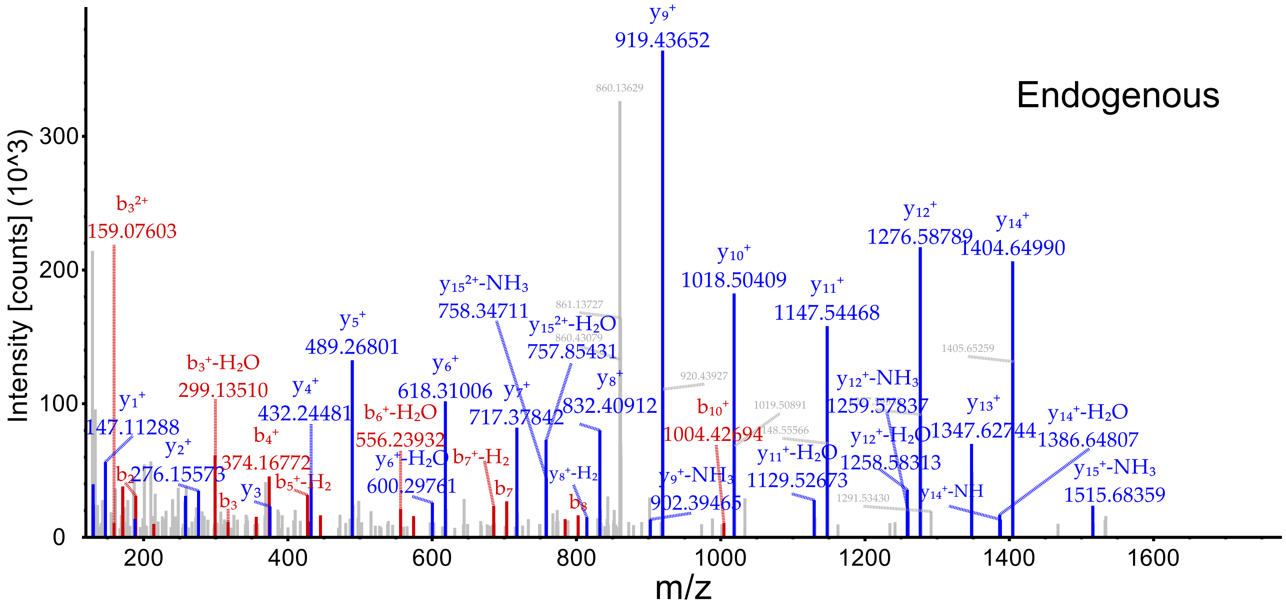

ZTT-1.raw #24966 RT: 35.0748 min
FTMS, 861.4008@hcd30.00, z=+2, Mono m/z=860.89785 Da, MH+=1720.78843 Da, Match Tol.=0.02 D

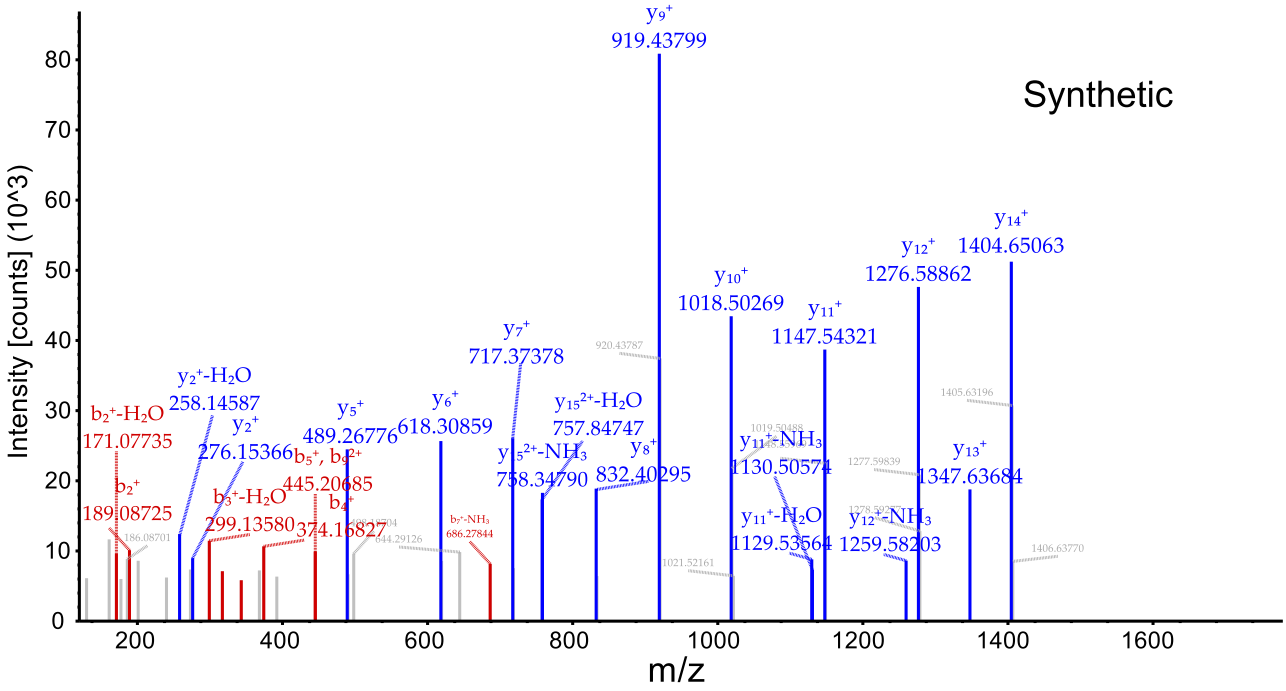

IP_613981_ KSVPSTGGVKKPHR

PYZ-1_1.raw #6000 RT: 12.3890 min
FTMS, 369.9197@hcd30.00, z=+4, Mono m/z=370.22025 Da, MH+=1477.85917 Da, Match Tol.=0.02 D

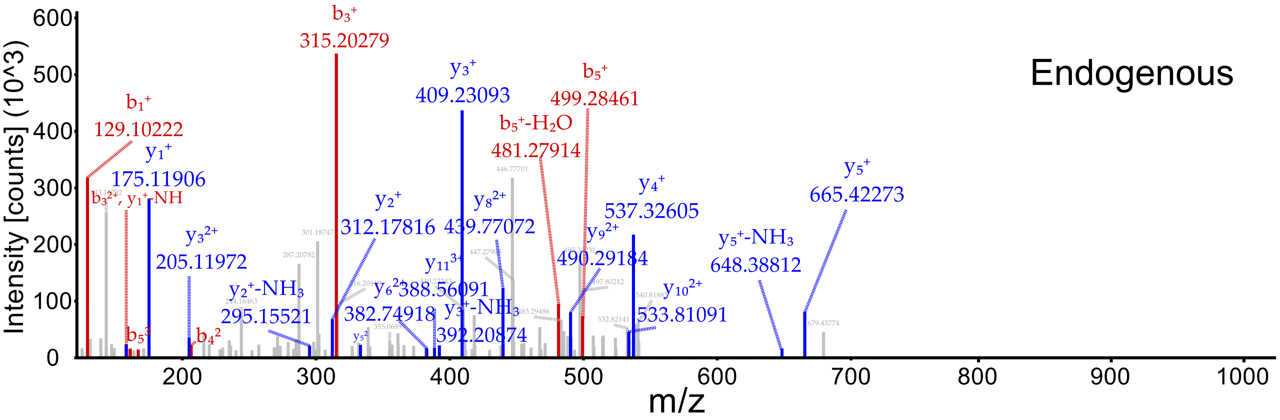

ZTT-3.raw #4967 RT: 11.3236 min
FTMS, 369.5541@hcd30.00, z=+4, Mono m/z=370.22078 Da, MH+=1477.86128 Da, Match Tol.=0.02 D

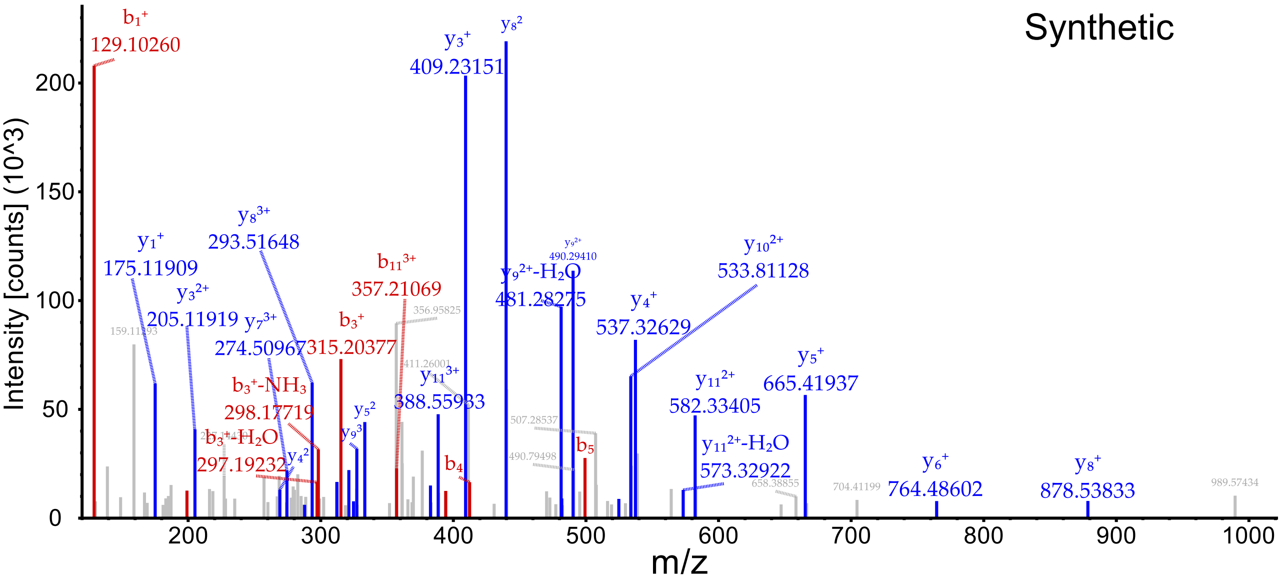

IP_603079_ LNEEASEEILK

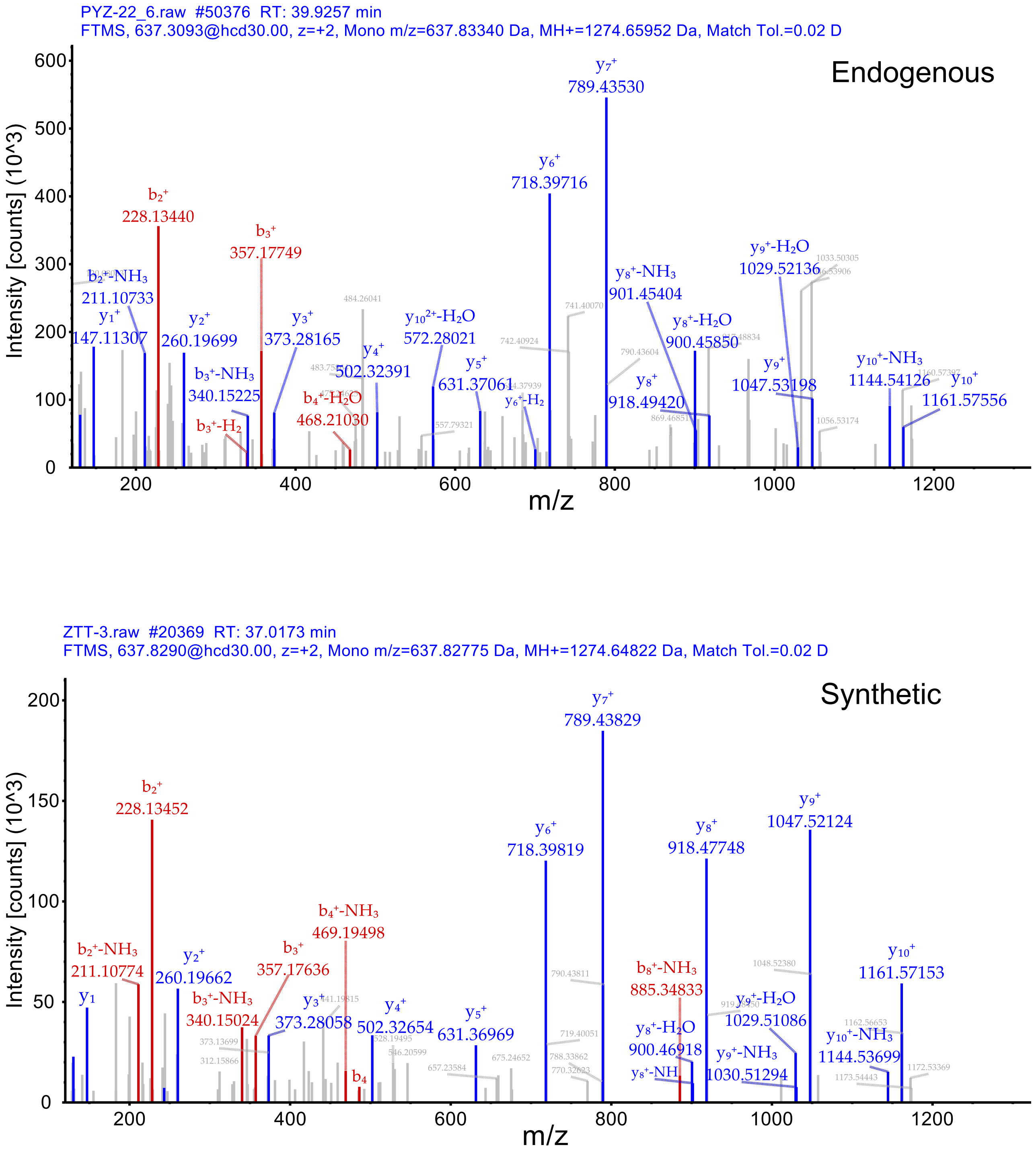

IP_620044_ QANLYISEGLHPR

PYZ-2_6.raw #53786 RT: 45.5102 min
FTMS, 749.3935@hcd30.00, z=+2, Mono m/z=749.39218 Da, MH+=1497.77708 Da, Match Tol.=0.02 D

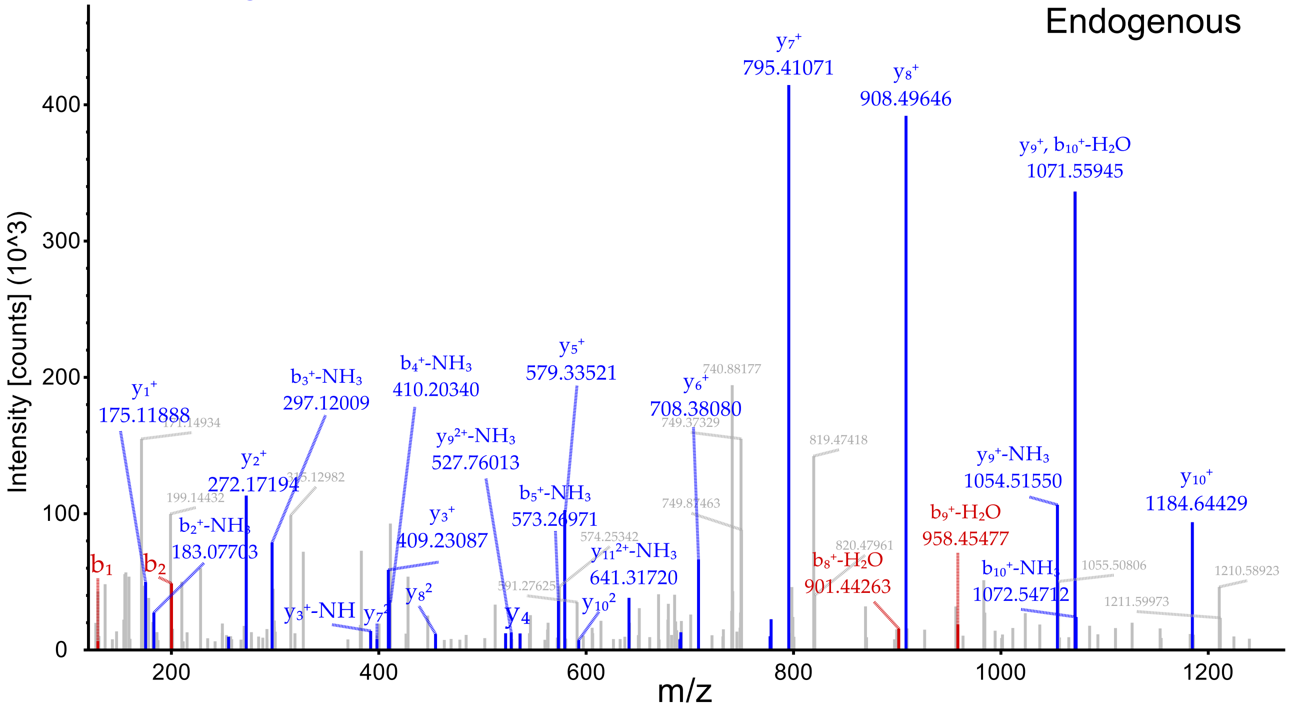

ZTT-1.raw #32639 RT: 46.7697 min
FTMS, 499.2591@hcd30.00, z=+3, Mono m/z=499.93126 Da, MH+=1497.77924 Da, Match Tol.=0.02 D

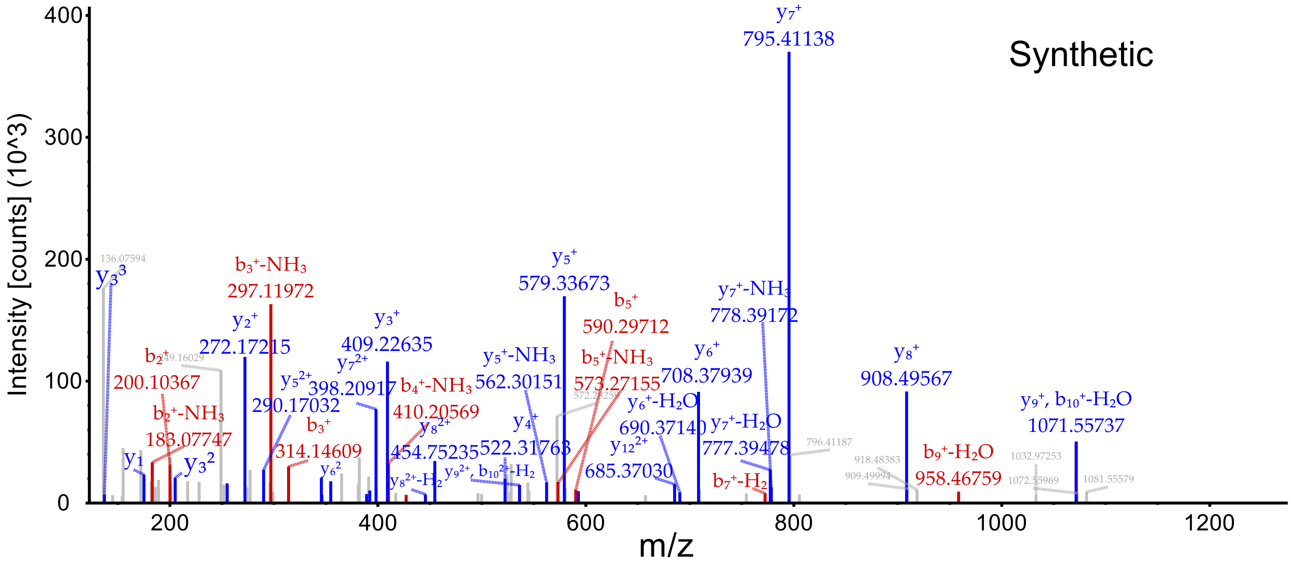

IP_767719_ ISTHLVIR

PYZ-13_3.raw #38624 RT: 34.9122 min
FTMS, 470.5531@hcd30.00, z=+2, Mono m/z=469.79197 Da, MH+=938.57667 Da, Match Tol.=0.02 D

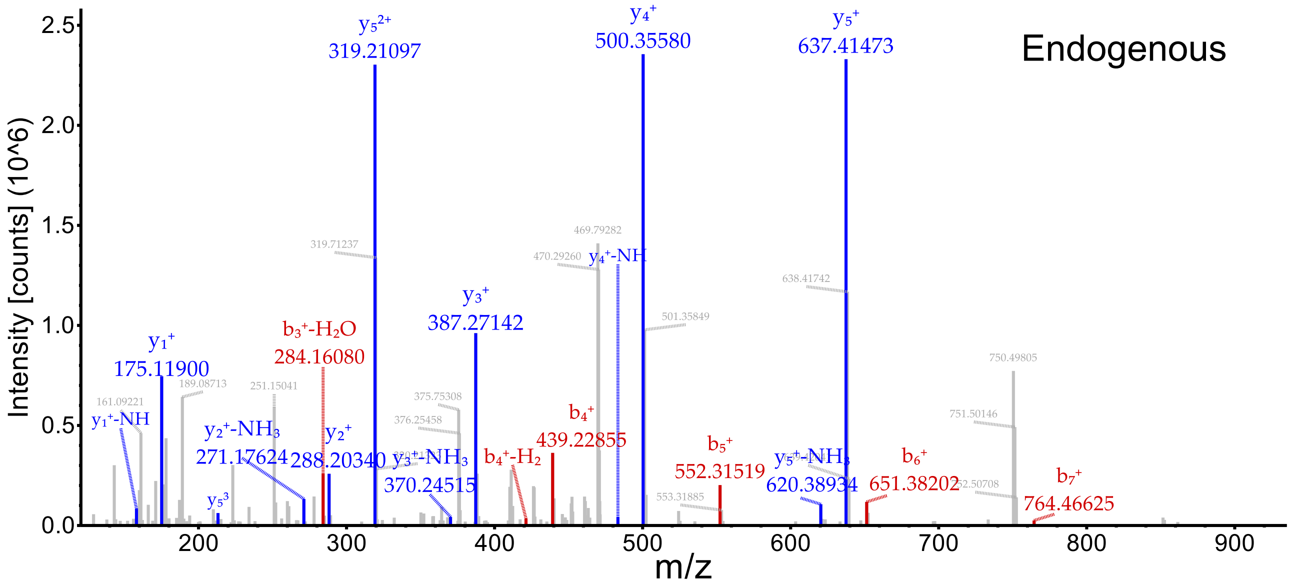

ZTT-2.raw #20430 RT: 35.8263 min
FTMS, 469.5793@hcd30.00, z=+2, Mono m/z=469.79272 Da, MH+=938.57817 Da, Match Tol.=0.02 D

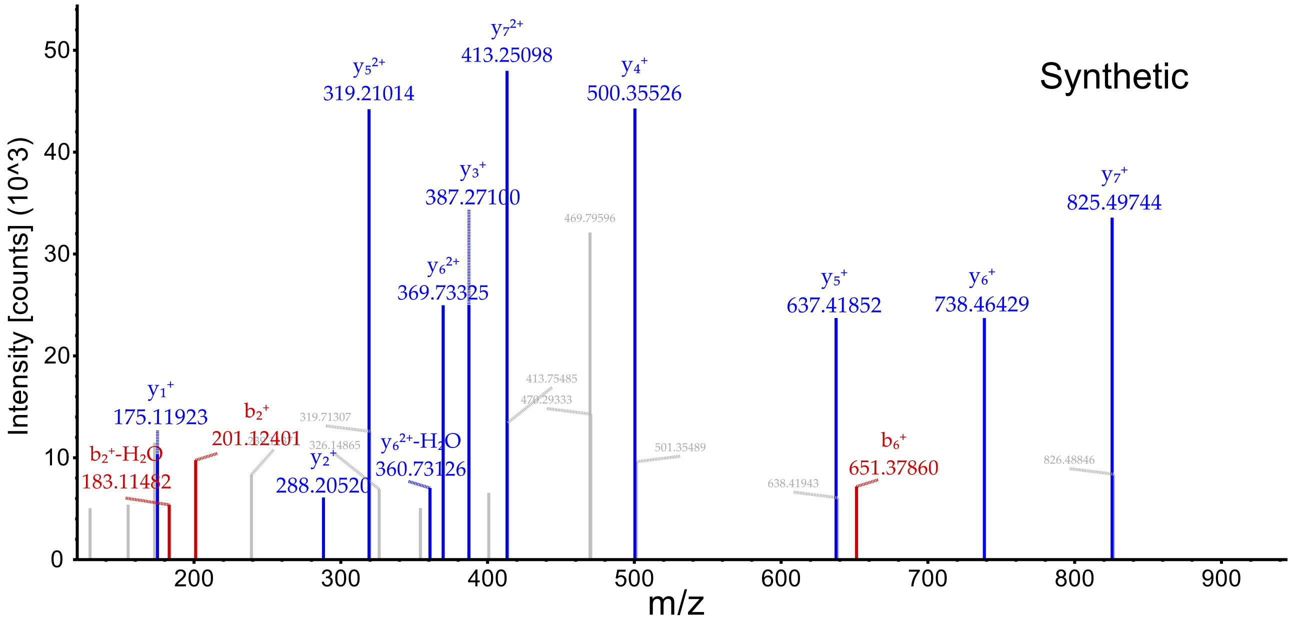

Supplementary Fig. S4 the MS spectrum of 10 synthetic peptides and its endogenous peptides
